# Complement-Related Proteins Control the Flavivirus Infection of *Aedes aegypti* by Inducing Antimicrobial Peptides

**DOI:** 10.1371/journal.ppat.1004027

**Published:** 2014-04-10

**Authors:** Xiaoping Xiao, Yang Liu, Xiaoyan Zhang, Jing Wang, Zuofeng Li, Xiaojing Pang, Penghua Wang, Gong Cheng

**Affiliations:** 1 Department of Basic Medical Sciences, School of Medicine, Tsinghua University, Beijing, People's Republic of China; 2 School of Life Sciences and Technology, Tongji University, Shanghai, People's Republic of China; 3 Section of Infectious Diseases, Yale University School of Medicine, New Haven, Connecticut, United States of America; 4 Collaborative Innovation Center for Diagnosis and Treatment of Infectious Diseases, Hangzhou, People's Republic of China; Washington University School of Medicine, United States of America

## Abstract

The complement system functions during the early phase of infection and directly mediates pathogen elimination. The recent identification of complement-like factors in arthropods indicates that this system shares common ancestry in vertebrates and invertebrates as an immune defense mechanism. Thioester (TE)-containing proteins (TEPs), which show high similarity to mammalian complement C3, are thought to play a key role in innate immunity in arthropods. Herein, we report that a viral recognition cascade composed of two complement-related proteins limits the flaviviral infection of *Aedes aegypti*. An *A. aegypti* macroglobulin complement-related factor (AaMCR), belonging to the insect TEP family, is a crucial effector in opposing the flaviviral infection of *A. aegypti*. However, AaMCR does not directly interact with DENV, and its antiviral effect requires an *A. aegypti* homologue of scavenger receptor-C (AaSR-C), which interacts with DENV and AaMCR simultaneously *in vitro* and *in vivo*. Furthermore, recognition of DENV by the AaSR-C/AaMCR axis regulates the expression of antimicrobial peptides (AMPs), which exerts potent anti-DENV activity. Our results both demonstrate the existence of a viral recognition pathway that controls the flaviviral infection by inducing AMPs and offer insights into a previously unappreciated antiviral function of the complement-like system in arthropods.

## Author Summary

Hosts are equipped with sophisticated machineries for detecting and eliminating invading viruses before they cause significant physiological damage. Unlike mammals which have both innate and adaptive immune systems, insects rely solely on the innate immune system to limit viral infection. Mosquitoes are natural vectors for many human pathogenic viruses, such as dengue virus (DENV) and yellow fever virus. Despite lacking an immunoglobulin-based humoral response, mosquitoes arm themselves with a functional complement-like system to ward off invading pathogens. Herein, we show that a system composed of complement-related factors recognizes and limits flaviviruses by inducing antimicrobial peptides expression. Understanding antiviral mechanisms in arthropods may provide novel strategies for limiting arboviral transmission in nature.

## Introduction

Animals have evolved multiple immune systems to eradicate pathogenic viruses. The innate immune response plays a crucial antiviral role in the early stage of infection. Immune responses are initiated based on the recognition of viral components, including viral surface glycoproteins and genetic material, by a group of recognition receptors. This recognition activates the complement system and intracellular antiviral signalling cascades, leading to the elimination of viruses and infected cells. Adaptive immunity is then evoked to produce virus-specific immunoglobulins for viral eradication. The antiviral machineries of blood-sucking arthropods, such as mosquitoes, ticks, and sand flies, are quite different from those of mammals [1]. Arthropods lack an immunoglobulin-based adaptive immune response [2]. Thus, the innate immune system of arthropods plays a central role in the antiviral processes of these organisms.

The complement system functions during the early phase of infection and directly mediates pathogen elimination [3]. The recent identification of complement-like factors in arthropods indicates that this system shares common ancestry as an immune defense mechanism in both vertebrates and invertebrates [4], [5], [6], [7]. Thioester (TE)-containing proteins (TEPs) share high similarity with mammalian C3 [8]. TEPs are generally divided into 3 distinct families, including alpha-2-macroglobulins (A2Ms), C3/C4/C5 complement factors, and insect TEPs (iTEPs) [9]. An *Anopheles* TEP induced by *Plasmodium berghei* can bind and kill parasitic ookinetes [5]. Multiple iTEPs in *Anopheles* (AgTEP1, AgTEP3 and AgTEP4) [10] play a role in clearing bacteria via phagocytosis. *Drosophila* TEPs (DmTEP2 and DmTEP3) are required for efficient phagocytosis of Gram-positive or Gram-negative bacteria in S2 cells [11]. However, *TEPs* mutation in *Drosophila* did not significantly influence survival rate after different bacterial infection*s*, suggested *Drosophila* TEPs act redundantly or that their absence can be compensated by other components of the immune response [12]. Macroglobulin complement-related factors (MCRs), which are members of the iTEP family, are highly conserved in metazoans and form an independent branch separate from other iTEP subfamilies in the phylogenetic tree. RNA interference screening has identified a *Drosophila* MCR (known as DmTEP6) that acts as a key factor in the efficient phagocytosis of *Candida albicans* [11]. Overall, the TEP-based complement-like system functions as an evolutionarily conserved anti-microbial mechanism in arthropods.

Scavenger receptors (SR) are cell surface glycoproteins defined by their ability to bind chemically modified low-density lipoproteins. These receptors are categorized into 6 classes, without conserved domains, suggesting that different primary amino acid (aa) sequences can encode similar 3-dimensional structures [13]. Several classes of SRs are located on the surface of immune cells and function as pathogen recognition receptors. SR-C, a membrane receptor identified in *Drosophila* that contains 2 complement control protein (CCP) domains (also designated Sushi repeat domains) in its extracellular region, is capable of recognizing both Gram-positive and Gram-negative bacteria and is thought to act as a pattern recognition receptor for phagocytosis [14]. The 2 CCP domains of *Drosophila* SR-C are thought to be responsible for microbial interaction.

The CCP domain is a signature module in many mammalian complement proteins. Each CCP consists of a domain of ∼60 aa residues, including a consensus pattern of 4 invariant cysteine residues and some additional conserved residues [15]. CCP has been shown to mediate the protein-protein interactions of complement components and to interact with pathogenic microorganisms. For example, complement receptor 2, containing 16 CCP repeats, acts as a receptor for the cleaved products of C3. Complement receptor 2 is also a well-known cellular entry receptor for Epstein-Barr virus in humans, and its CCP-1 and -2 domains are required for Epstein-Barr virus binding [16]. A membrane cofactor protein (MCP) with 4 CCP repeats has been demonstrated to function as a cellular receptor for measles virus [17]. Structural analysis has shown that the CCP-1 and -2 domains of MCP are responsible for measles virus recognition [15]. Another complement regulator with 20 CCP repeats, factor H, reportedly binds the human immunodeficiency virus surface glycoproteins gp41 and gp120 [18], [19].

Many mosquito-transmitted flaviviruses, such as West Nile virus, Japanese encephalitis virus, dengue virus (DENV) and yellow fever virus (YFV), are etiologic agents of severe human diseases, including hemorrhagic fever, biphasic fever, encephalitis, and meningitis. DENV is maintained in a transmission cycle between humans and *Aedes* mosquitoes [20], [21]. Four serotypes of DENV, sequentially designated DENV1-4, exist in nature [22]. *Aedes aegypti*, a member of the *Culicinae* subfamily, shows a close association with human populations and is a primary vector for DENVs [23]. Because *A. aegypti* is easy to cultivate in the laboratory and its genome has been well characterized [24], [25], it has become an ideal model for the investigation of flaviviral pathogenesis and innate immune responses in invertebrates [7], [26], [27], [28]. Herein, we report that an *A. aegypti* MCR (AaMCR) is a crucial effector in opposing the flaviviral invasion of mosquitoes. Furthermore, we identified an SR-C with 2 CCP domains in *A. aegypti* that efficiently recognizes DENV and recruits AaMCR to stimulate the expression of antimicrobial peptides (AMPs). Our findings suggest a new pattern recognition receptor pathway that senses flaviviruses and initiates an antiviral cytokine-like response in *A. aegypti.*

## Results

### Phylogenetic analysis of MCR factors in *A. aegypti*

**Figure 1:**
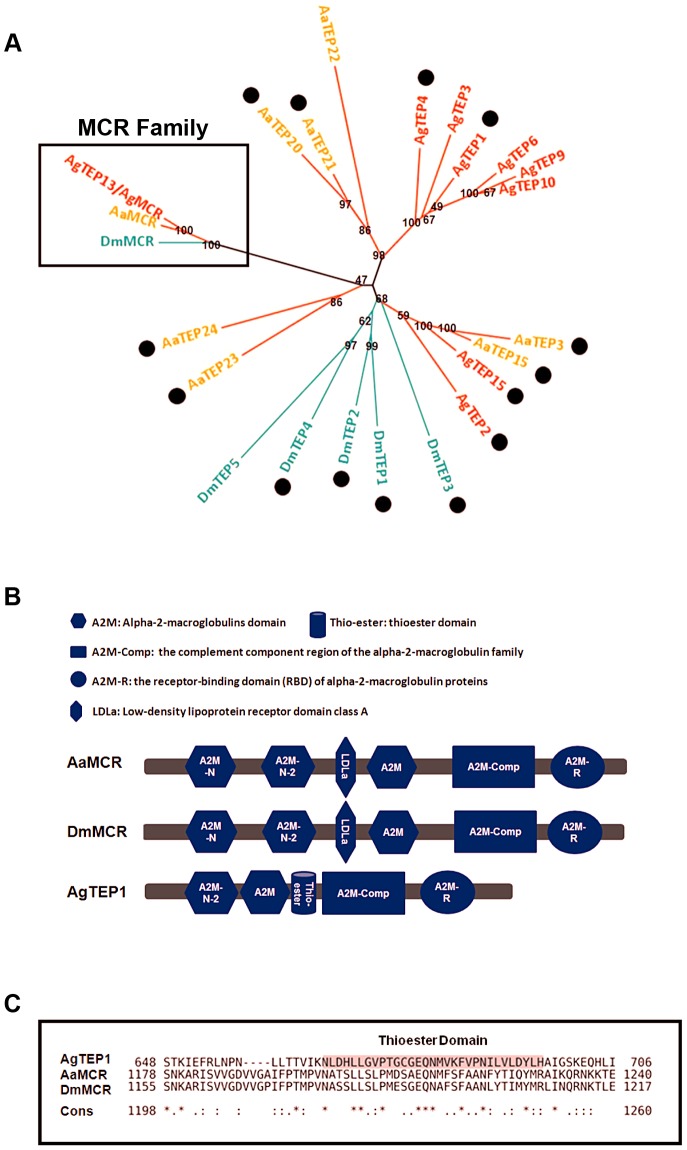
Comparison of the functional domains and phylogenetic analysis of insect thioester-containing proteins (iTEPs). (A) Unrooted phylogenetic tree of iTEPs. The tree was constructed using the neighbour-joining (NJ) method based on the alignment of 23 iTEP protein sequences. The bootstrap values of 500 replicates (%) are indicated on the branch nodes. *Anopheles gambiea* (Ag), *Aedes Aegypti* (Aa) and *Drosophila melanogaster* (Dm) are indicated with red, yellow and green, respectively. Period represents the iTEPs with thioester domain. (B) Schematic representation of AaMCR, DmMCR and AgTEP1. The functional modules of MCRs and TEPs were predicted using the SMART (http://smart.embl-heidelberg.de/smart/set_mode.cgi?GENOMIC=1) and Pfam websites (http://pfam.sanger.ac.uk/). (C) Alignment of the sequence of thioester domain using CLUSTAL-X. The shadowed sequence is the predicted thoiester domain. Asterisk indicates the identical residues in all sequences of the alignment; colon indicates the conserved substitutions; period indicates the semi-conserved substitutions.

The proteins of the TEP superfamily are key constituents of the immune systems of both vertebrates and invertebrates. TEPs are generally divided into 3 distinct subfamilies: complement C3/C4/C5, A2Ms, and iTEPs. Previous studies have suggested that several iTEP genes found in *Anopheles* and *Drosophila* play a role in microbial elimination [10], [11]. Furthermore, silencing an *A. aegypti TEP* homologue (*AAEL012267*, known previously as *AeTEP1*) significantly enhances flaviviral infections [7], indicating a potential role of *iTEPs* in combating the viral infection of insects. Given the complicated immune function of *iTEPs*, we analyzed the phylogenetic relationships of iTEP proteins among *Anopheles gambiae*, *A. aegypti*, and *Drosophila melanogaster*. MCRs, characterized as a subfamily of iTEPs, cluster along a single branch (Figure 1A). *AAEL012267* is grouped into the MCR family, indicating that it is an *A. aegypti* MCR homologue (see Figure 1A).The *AAEL012267*-encoded protein is predicted to contain domains that are identical to those of *Drosophila* MCR (DmMCR), suggesting possible conserved functions of these proteins (Figure 1B). We have therefore re-designated *AAEL012267* as *AaMCR* throughout this study. The thioester domain of iTEPs binds the surface of microbes via a covalent bond and triggers the phagocytosis and opsonization of microbes. However, not all TEPs contain the TE module (Figure 1C). The lack of this domain in MCRs suggests a distinct mechanism of action.

### AaMCR resists the flaviviral infections of *A. aegypti*

**Figure 2:**
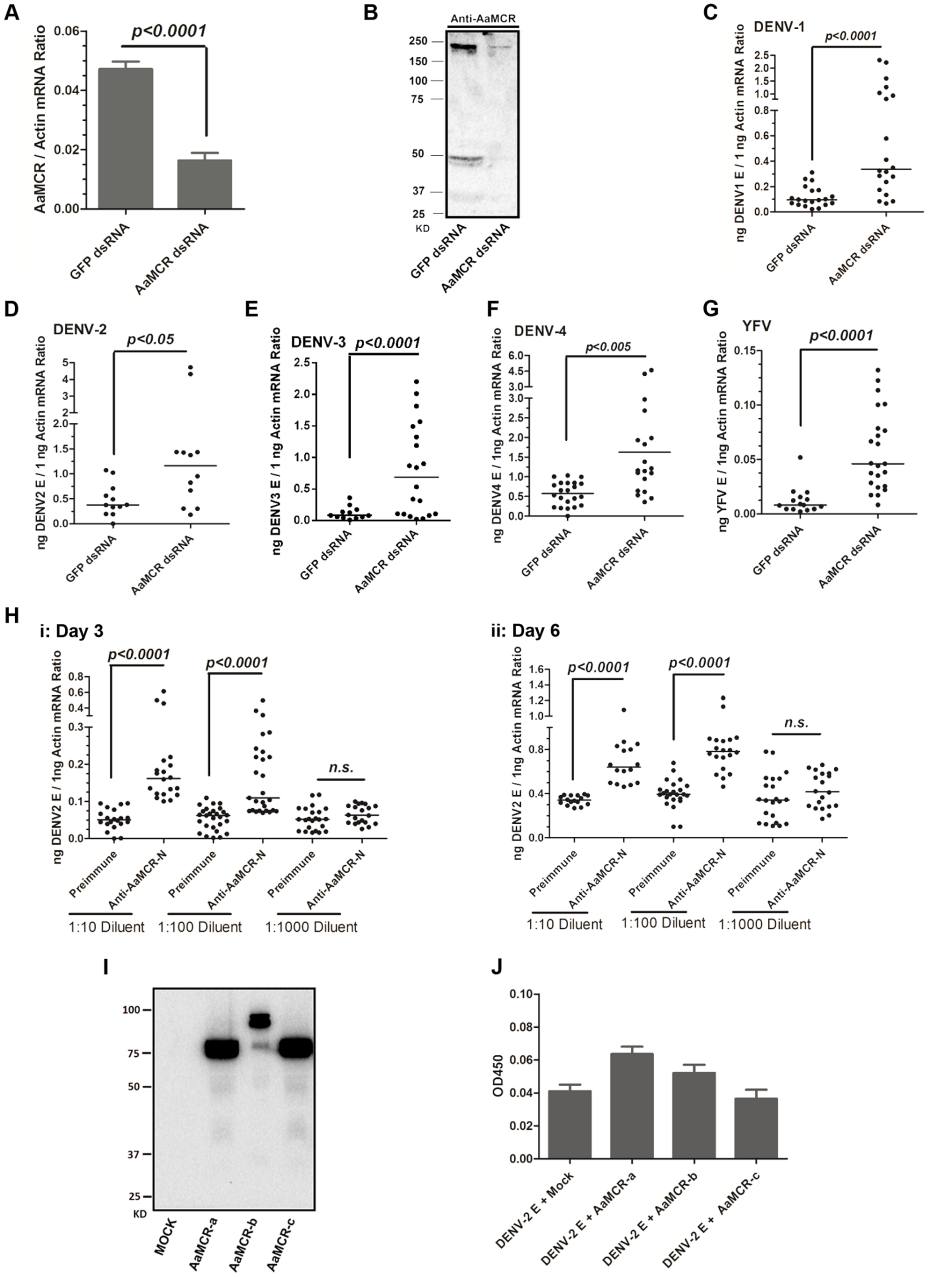
AaMCR restricts flaviviral infections. (A-B) Knockdown of the *AaMCR* gene. The mosquitoes were microinjected with *AaMCR* or *GFP* dsRNA. The inoculated mosquitoes were sacrificed, and *AaMCR* abundance was assessed via qPCR (A) and immuno-blotting with an AaMCR antibody (B) at 6 days post microinjection. 50 μg of protein from mosquito lysates was loaded into each lane (B). (C-G) Silencing of *AaMCR* enhanced DENVs and YFV (17D) infections in *A. aegypti*. 10 MID_50_ DENVs or YFV was inoculated at 3 days post-*AaMCR* silencing. The viral load was assessed at 6 days post-infection through qPCR and normalized against *A. aegypti actin* (*AAEL011197*). The primers and probes used for qPCR are described in Table S2. The experiment was repeated three times with similar results. One dot represents 1 mosquito and the horizontal line represents the median of the results. The data were analyzed statistically by the non-parametric *Mann-Whitney* test. (H) Immuno-blockade of AaMCR enhanced the DENV-2 infection of *A. aegypti*. The AaMCR-N antibody, in 10-fold serial dilutions, was premixed with 10 MID_50_ DENV-2 for thoracic co-microinjection. The treated mosquitoes were sacrificed to examine the viral load at 3 (i) and 6 (ii) days post-infection via qPCR and normalized against *A. aegypti actin*. The results were pooled from 3 independent experiments. One dot represents 1 mosquito and the horizontal line represents the median of the results. The data were analyzed statistically using the non-parametric *Mann-Whitney* test. (I) The expression of AaMCR fragment peptides in *Drosophila* S2 cells. Three fragments of the AaMCR (a, 30–601 aa; b, 590–1,240 aa; c, 1,200–1,793 aa) with a C-terminal HA tag were cloned into the pMT/BiP/V5-His A vector and expressed in the S2 cell supernatant. The supernatant from empty vector-transfected S2 cells was used as a mock. The recombinant peptides were detected with an anti-HA antibody via western blotting. (J) The fragments of AaMCR do not directly interact with DENV-2 E protein in ELISA. The cell supernatant, including the AaMCR fragments were collected at 48 hrs after transfection. The DENV-2 E protein was expressed and purified from a *Drosophila* expression system. Each plate well was coated with 2 ug of DENV-2 E. The interaction was determined using an anti-HA antibody. The empty DNA vector-transfected S2 cell supernatant was used as a negative control. The data are expressed as the mean ± standard error from 3 independent experiments.

To examine the physiological role of AaMCR in flaviviral infection, we knocked down *AaMCR* using double-stranded RNA (dsRNA) via thoracic inoculation. The expression of *AaMCR* was significantly decreased at both the mRNA (Figure 2A) and protein (Figure 2B) levels following dsRNA treatment. Three days after gene silencing, 4 DENV serotypes and YFV were microinjected into the mosquitoes, and their viral loads were assessed 6 days post-infection via quantitative polymerase chain reaction (qPCR) and plaque assay. Consistent with our previous results [7], AaMCR knockdown significantly increased the levels of DENV 1–4 and YFV transcripts (Figure 2C–2G) and the number of virions (Figure S1A–S1E), confirming an important antiviral function of *AaMCR* in mosquitoes.

To understand the role of AaMCR in viral infections more thoroughly, we expressed and purified an N-terminal peptide of from AaMCR (AaMCR-N, 30–601 aa) from *Escherichia coli* (Figure S1F) and generated mouse polyclonal antiserum against this fragment. The antibody recognized a full-length AaMCR of ∼200 kDa and a smaller band of ∼50 kDa in mosquito lysates (Figure S1G) and hemolymph (Figure S1H). To validate the above results, we generated rabbit polyclonal antiserum using 2 synthetic peptides from the N-terminus of AaMCR. The same bands of ∼200 kDa and ∼50 kDa were detected in a mosquito lysates (Figure S1I). These results suggest that AaMCR is likely constitutively and rapidly processed into small active fragments and its cleaved peptides function in soluble form in the hemolymph of *A. aegypti*. To rule any off-target effects dsRNA-mediated RNA interference and validate the function of AaMCR, we premixed serial dilutions of the AaMCR-N antibody with the DENV-2 virus and thoracically microinjected the mixture into *A. aegypti*. Blocking the function of AaMCR using this specific antibody dramatically enhanced DENV-2 infectivity in mosquitoes at both 3 days (Figure 2H, i) and 6 days (Figure 2H, ii) post-inoculation.

Previous reports have demonstrated a direct interaction between iTEPs and pathogens via a TE bond. *Anopheles* TEP1 kills *P. berghei* ookinetes by binding to their surface proteins [5]. We therefore assessed the interaction between AaMCR and DENV-2. AaMCR is a 1,793-aa protein comprised of multiple complement-like domains but lacks an internal TE domain (Figure 1B). According to the predicted domains, we divided AaMCR into 3 fragments (AaMCR-a, -b, -c; Figure S1J) to be expressed in *Drosophila* S2 cells (Figure 2I). None of the 3 segments of AaMCR interacted directly with the DENV-2 envelope (E) protein in enzyme-linked immunosorbent assays (ELISAs) (Figure 2J) or co-immunoprecipitation (co-IP) assays (data not shown). We also assessed *AaMCR* mRNA expression in various mosquito tissues. We noted that the abundance of *AaMCR* mRNA varied among the tissues, with the highest abundance being found in the salivary glands, followed by the hemocytes and midgut. However, DENV-2 infection did not induce *AaMCR* mRNA expression in the investigated tissues at serial time points (Figure S1K).

### Proteins containing CCP domains restrict the flaviviral infections of *A. aegypti*

The CCP domain is an evolutionarily conserved module in the complement system that is essential for reactions in the complement cascade. AaMCR shares high homology with mammalian C3/C4/C5 complement factors. The cleaved fragments of C3 and C4 are recognized by the CCP domains of the complement receptors [29]. However, the CCP domains of several membrane proteins can be manipulated by microbial pathogens to be used as receptors for cellular entry [16], [17]. Our results show that AaMCR does not bind directly to DENV-2, indicating that adaptor molecules may exist to facilitate DENV recognition by AaMCR. We therefore speculated that proteins with CCP domains found in *A. aegypti* may play a role in the AaMCR-mediated antiviral response.

**Figure 3:**
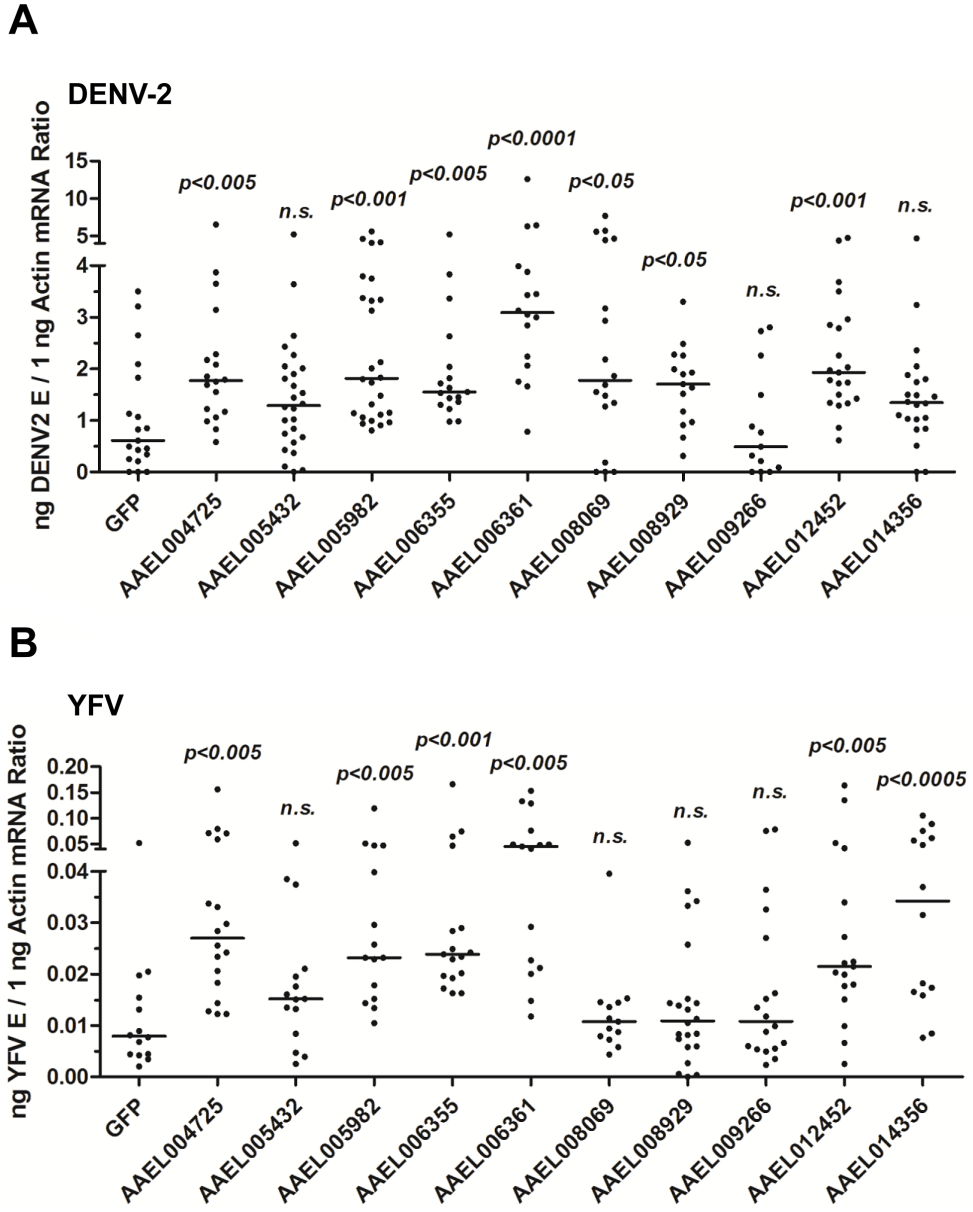
The role of CCP domain-containing proteins in flaviviral infection. Each *CCP* gene was silenced through thoracic microinjection of dsRNA and the effect on the viral burden was assessed on 6 days post-DENV-2 (A) or YFV (B) infection. *GFP* dsRNA-inoculated mosquitoes served as mock controls. The viral loads were measured via qPCR, and normalized against *A. aegypti actin*. One dot represents 1 mosquito and the horizontal line represents the median. The data were analyzed statistically using the non-parametric *Mann-Whitney* test. The results were combined from 2 independent experiments.

To test this hypothesis, we identified a family of 10 proteins with CCP domains in *A. aegypti* (Table S1). These 10 *CCP* genes were knocked down individually via thoracic microinjection of dsRNA. DENV-2 was then inoculated into the mosquitoes, and the resultant viral loads were quantified through qPCR 6 days after infection. Individual knockdown of 7 *CCP* genes in mosquitoes significantly enhanced the DENV-2 burden compared with that found in green fluorescent protein (*GFP*) dsRNA-treated mosquitoes (Figure 3A). Moreover, the silencing of 6 *CCP* genes increased mosquito susceptibility to YFV (17D vaccine strain) infection (Figure 3B), indicating a general antiviral role for the *CCP* genes of *A. aegypti*. Among all of the identified family members, *AAEL006361* exhibited the most significant effect on DENV-2 infection. Viral loads were enhanced by ∼4 folds in *AAEL006361* dsRNA-treated mosquitoes compared with the *GFP* dsRNA group (see Figure 3A and 3B). *AAEL006361* was effectively silenced at both the mRNA (Figure S2A) and protein (Figure S2B) levels, indicating that the enhancement of DENV infection was correlated with dsRNA inoculation. Similar results were observed for other DENV serotypes (Figure S2C and S2D).

### AAEL006361 is an SR that recognizes flaviviruses *in vitro* and *in vivo*

**Figure 4:**
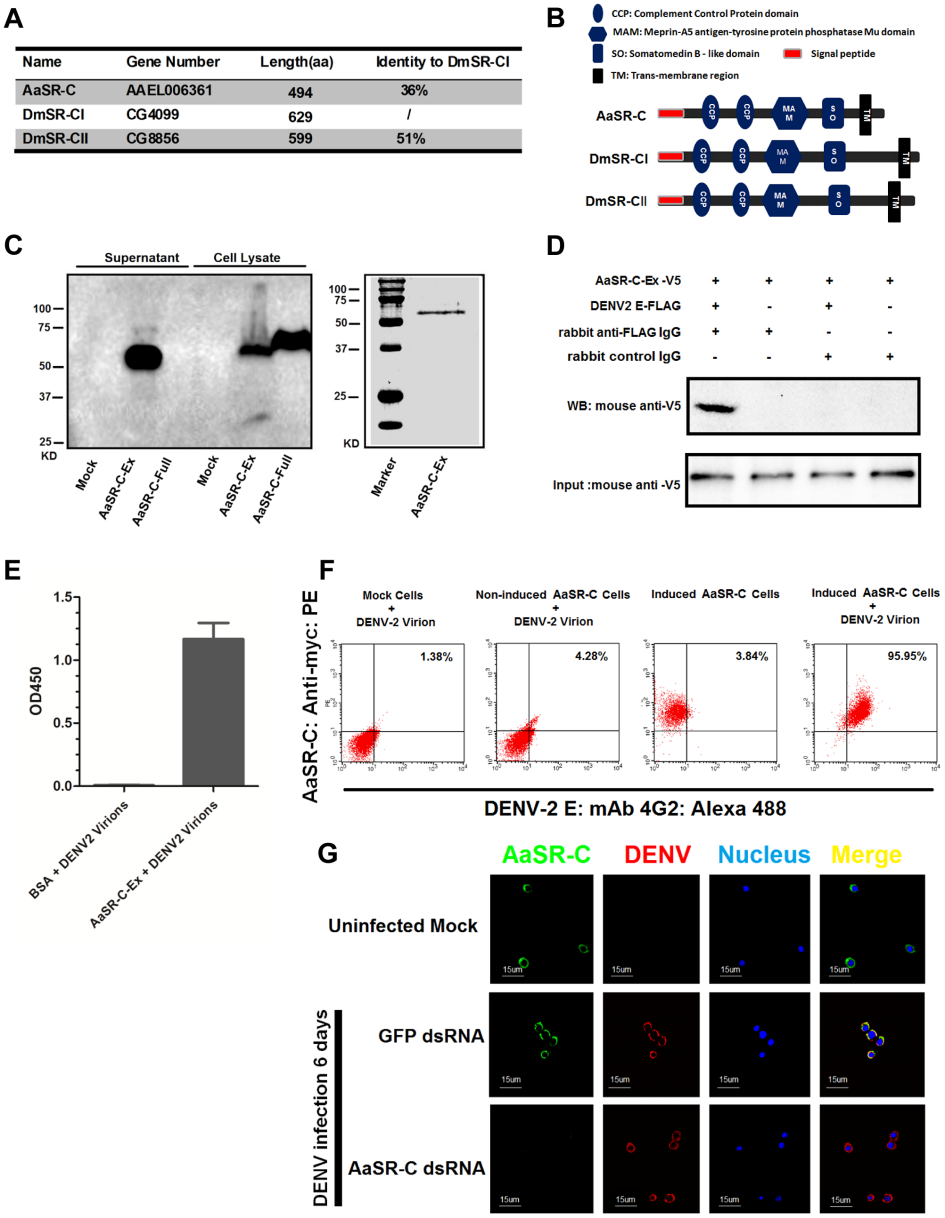
A Scavenger receptor-C with CCP domains recognizes DENV-2 *in vitro* and *in vivo*. (A) Percentage of amino acid identity of insect SR-Cs. (B) Schematic representation of SR-Cs in *A. aegypti* and *D. melanogaster*. The functional modules were predicted in SMART (http://smart.embl-heidelberg.de/smart/set_mode.cgi?GENOMIC=1) and Pfam websites (http://pfam.sanger.ac.uk/). (C) Expression and purification of AaSR-C from *Drosophila* S2 cells. The extracellular region (AaSR-C-Ex) or full length (AaSR-C-Full) *AaSR-C* was cloned into the pMT/BiP/V5-His-A expression vector. The recombinant plasmids were transfected into *Drosophila* S2 cells, and their expression was probed using an anti-V5 mAb. The supernatant or lysates from mock-transfected S2 cells was used as the mock control (Left panel). Recombinant AaSR-C-Ex, produced in *Drosophila* cells, was purified using an Ni-His column (Right panel). (D) AaSR-C-Ex interacted with DENV-2 E proteins in co-immunoprecipitation assay. Purified AaSR-C-Ex (V5) and DENV-2 E (FLAG) were used to investigate the interaction of the proteins. Control rabbit IgG was used as a mock control to exclude non-specific interactions. The protein complex was pulled down with a rabbit anti-FLAG antibody and detected using a mouse anti-V5 antibody. We reproduced the experiment 3 times. (E) AaSR-C-Ex captured DENV-2 virions in an ELISA. Binding was probed using the flavivirus E mAb 4G2. The data are presented as the mean ± standard error. The experiment was reproduced 3 times. (F) AaSR-C bound DENV-2 virions on the cell surface. A Cu^2+^-inducible stable S2 cell line was generated to express the full-length AaSR-C. DENV-2 virions were incubated with AaSR-C expressing cells at 4°C for 1 hr. Non-induced stable cells and empty vector-transfected S2 cells containing virions served as the mock control groups. The interaction between AaSR-C and DENV was measured through flow cytometry. The DENV virions were stained using the flaviviral E mAb 4G2 and anti-mouse IgG Alexa-488; AaSR-C was probed using a Myc mAb and anti-rabbit IgG Phycoerythrin (PE). The data was analyzed using FlowJo software. The presented data was representative of 3 independent experiments with similar results. (G) The *in vivo* association between AaSR-C and DENV-2 in *A.aegypti* hemocytes. Hemolymph was collected from uninfected mosquitoes, *AaSR-C* silenced infected mosquitoes and *GFP* dsRNA treated infected mosquitoes to undergo immunofluorescence staining. AaSR-C was stained with anti-rabbit IgG Alexa-488 (Green), and the DENV-2 E protein was identified using anti-mouse IgG Alexa-546 (Red). Nuclei were stained blue with To-Pro-3 iodide (Blue). Images were examined using a Zeiss LSM 780 meta confocal 63×objective lens.

*AAEL006361* encodes a trans-membrane protein with a signal peptide that contains 2 CCP domains in its extracellular region. Sequence analysis that *AAEL006361* is a homologue of *Drosophila* SR-C (*DmSR-C*; Figure 4A), and the protein encoded by *AAEL006361* presents the same highly conserved domains as DmSR-C (Figure 4B). We therefore designated *AAEL006361* as *A. aegypti* SR-C (*AaSR-C*) in this study. To detect the endogenous expression of AaSR-C, we generated a rabbit polyclonal anti-AaSR-C antibody against the extracellular region. A ∼50-kDa protein band was detected in mosquito lysates (Figure S3A), and its size corresponded to the predicted full length of AaSR-C. We therefore cloned and expressed the extracellular region of *AaSR-C* (*AaSR-C-Ex*, 23–400 aa) in a *Drosophila* expression system. The recombinant peptide was then purified and detected through immunoblotting (Figure 4C, left panel) and Coomassie Blue staining (Figure 4C, right panel).

To understand the antiviral mechanism of AaSR-C, we next assessed whether AaSR-C binds directly to DENV-2. The purified AaSR-C-Ex peptide interacted strongly with the DENV-2 E protein in a co-IP assay (Figure 4D). Purified AaSR-C-Ex also captured DENV-2 virions efficiently (Figure 4E). AaSR-C was predicted to be a membrane protein (Figure 4B). To verify that DENV virions bind AaSR-C on the cell surface, we cloned and expressed full-length *AaSR-C* with its trans-membrane region (*AaSR-C-Full*, 23–494 aa; see Figure 4C, left panel) and constructed a Cu^2+^-inducible AaSR-C-expressing stable cell line. AaSR-C-expressing cells were incubated with DENV-2 virions, and their association with the cell surface was examined using flow cytometry. Non-induced stable cells and empty vector-transfected S2 cells were included as controls. More than 90% of the AaSR-C-expressing cells were bound by DENV-2 virions and showed double-positive staining compared with a negligible number of double-stained cells that were observed in the controls (Figure 4F). We next performed immunofluorescence staining to explore the location of native AaSR-C and DENV-2 virions. AaSR-C was highly expressed on the surface of hemocytes (Figure 4G, the 1st row). Following *AaSR-C* silencing, the polyclonal antibody against AaSR-C did not detect endogenous AaSR-C expression on hemocytes (Figure 4G, the 3rd row), indicating the high specificity of the antibody. Overlapping localization of AaSR-C and DENV was observed on mosquito hemocytes (Figure 4G, the 2nd row), suggesting that both AaSR-C and DENV locate to function on the cell surface. In agreement with the immunofluorescence staining results, *AaSR-C* transcripts were found to be more highly expressed in hemocytes compared with salivary glands and the midgut (Figure S3B). We also noted that *AaSR-C* mRNA expression remained unchanged after DENV-2 infection (Figure S3B).

### AaSR-C acts in conjunction with AaMCR in a pathway that opposes DENV infection

**Figure 5:**
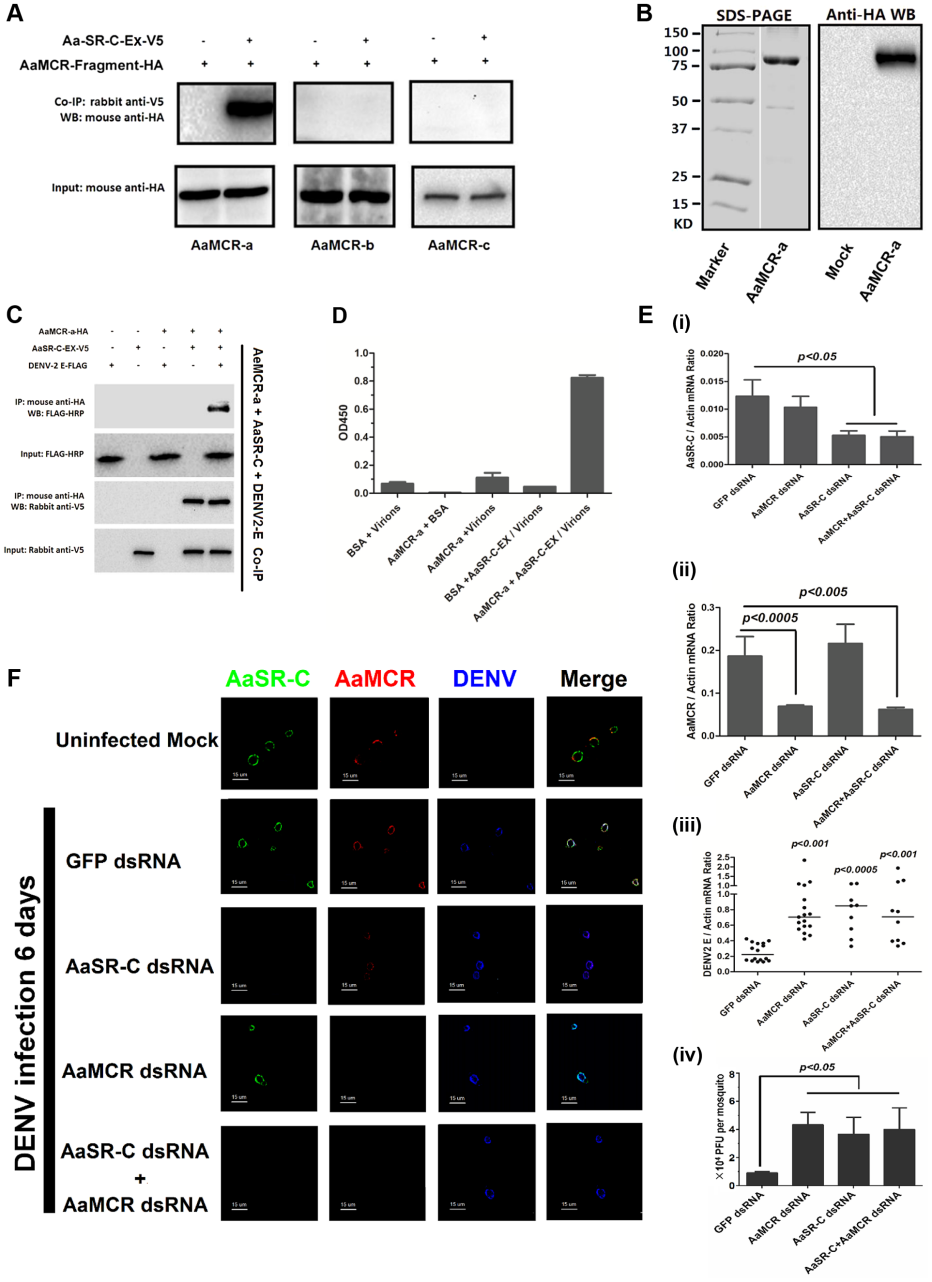
AaMCR and AaSR-C function in a pathway that opposes DENV-2 infection. (A) The interaction between 3 AaMCR fragments and AaSR-C in co-IP assays. Three *AaMCR* gene fragments were cloned into the pMT/BiP/V5-His A vector. The recombinant plasmids were transiently transfected into S2 cells. The cell supernatant was used for investigation of the AaMCR/AaSR-C interaction. The protein complex was pulled down with a rabbit anti-V5 antibody and probed using a mouse anti-HA antibody. The experiment was reproduced 3 times. (B) Expression and purification of AaMCR-a in *Drosophila* S2 cells. The purified AaMCR-a was separated through SDS-PAGE (Left Panel) and detected with an anti-HA antibody via western blotting (Right Panel). The supernatant from empty vector-transfected S2 cells was used as the mock control. (C) AaSR-C-Ex acted as an adaptor in the interaction between the AaMCR-a and DENV-2 E proteins. The purified AaSR-C-Ex, AaMCR-a and DENV-2 E proteins were mixed and pulled down with a mouse anti-HA antibody (AaMCR-a) and detected using a rabbit anti-V5 antibody (AaSR-C) and anti-FLAG-HRP antibody (DENV-2 E). The experiment was repeated 3 times with similar results. (D) AaSR-C-Ex connected AaMCR-a to DENV-2 virions. Purified AaMCR-a or BSA was pre-coated into the ELISA plate wells. DENV-2 virions either mixed with AaSR-C-Ex or without AaSR-C-Ex were added to the protein-coated wells. The signal was detected using the flavivirus E mAb 4G2. The data are expressed as the mean ± standard error. The experiment was reproduced by 3 times with similar results. (E) Double knockdown of *AaMCR* and *AaSR-C* showed similar effects on DENV-2 infection to individual knockdown. Both *AaSR-C* (i) and *AaMCR* (ii) were knocked down using a dsRNA mixture in the *AaSR-C*/*AaMCR* co-silenced group. DENV-2 replication and the numbers of infectious DENV-2 virions in the mosquitoes were measured via qPCR (iii) and plaque assays (iv). Statistical analysis was performed using the non-parametric *Mann-Whitney* test. The data on gene silencing (i, ii) and from plaque assays (iv) are expressed as the mean ± standard error. The horizontal line depicts the median (iii). Each dot represents an individual mosquito. The result was representative of 3 independent experiments. (F) Immunostaining of AaMCR, AaSR-C and DENV-2 in *A. aegypti* hemocytes. The hemocytes were dissected from uninfected mosquitoes, *AaMCR* and/or *AaSR-C* silenced infected mosquitoes and *GFP* dsRNA treated infected mosquitoes at 6 days post-infection. AaSR-C was stained with anti-rabbit IgG Alexa-488 (Green); AaMCR was probed using anti-mouse IgG Alexa-546 (Red); the DENV-2 E protein was probed with DENV-2 human antiserum (purified IgG) and anti-human IgG Alexa-633 (Blue). Images were examined using a Zeiss LSM 780 meta confocal 63×objective lens.

In the mammalian complement system, several complement proteins have CCP-mediated interaction with C3 and C4 cleaved fragments. In the present study, we assumed that AaSR-C may also interact with AaMCR in the process of viral elimination. Three fragments (AaMCR-a to AaMCR-c) arranged from the N- to C-terminus were expressed in *Drosophila* S2 cells (see Figure 2I). The binding of AaMCR segments to AaSR-C was then determined through co-IP assays. AaSR-C interacted strongly with the N-terminal of AaMCR-a (30-601 aa). However, no interaction was observed between AaSR-C and AaMCR-b (590–1,240 aa) or AaMCR-c (1,200–1,793 aa) (Figure 5A). We next expressed and purified AaMCR-a in a *Drosophila* expression system (Figure 5B, left panel). The purification of the protein was confirmed through western blotting using an anti-HA tag monoclonal antibody (see Figure 5B, right panel). The purified AaMCR-a interacted with AaSR-C-Ex in an ELISA (Figure S4A) and a co-IP assay (Figure S4B).

We have demonstrated crucial roles for both AaSR-C and AaMCR in controlling the DENV-2 infection of *A. aegypti* in addition to a direct interaction of AaSR-C with DENV-2 or the N-terminus of AaMCR. We next examined whether AaSR-C and AaMCR cooperate in a pathway that opposes DENV infection. As shown in Figure 5C, purified DENV-2 E, AaSR-C-Ex, and AaMCR-a formed a complex in co-IP assays, whereas AaMCR-a did not bind directly to the DENV-2 E protein (Figure 5C), suggesting that AaSR-C-Ex functions as a linker to bring the other 2 proteins together. Likewise, AaSR-C-Ex was shown to be necessary for AaMCR-a binding to DENV-2 virions in an ELISA (Figure 5D). To determine the relationship between AaSR-C and AaMCR in DENV infection, we co-silenced *AaSR-C* (Figure 5E, i) and *AaMCR* (see Figure 5E, ii) in mosquitoes using dsRNA. Double knockdown had the same effect on DENV replication (Figure 5E, iii) and the number of DENV-2 virions (Figure 5E, iv) as single knockdown, suggesting that *AaSR-C* and *AaMCR* function in the same pathway opposing the DENV infection of *A. aegypti*.

In the above experiments, the mosquitoes were infected through intra-thoracic inoculation. To further evaluate the dissemination of DENV in various mosquito tissues following the infection by oral feeding, we silenced *AaMCR* and *AaSR-C*, both individually and together, via intra-thoracic microinjection of dsRNA. Three days after the dsRNA treatment, the mosquitoes were fed with Vero cells-generated DENV-2 and fresh human blood and the specific tissues were dissected at serial time points to evaluate the kinetics of viral dissemination. In accordance with our previous results obtained through intra-thoracic infection (Figure 5E, iii), in the oral feeding experiment, silencing *AaMCR* and/or *AaSR-C* significantly enhanced DENV-2 burden in whole mosquitoes and various tissues, indicating that the complement-like system works as a general mechanism to limit viral infection in mosquitoes (Figure S5A-S5C). Moreover, the silencing of these complement components increased the number of infectious DENV-2 virions in salivary glands extracts (SGE) as determined in plaque assay (Figure S5D), suggesting that antiviral immunity in mosquitoes may influence their viral transmission capacity.

To understand the association between AaSR-C, AaMCR, and DENV more thoroughly *in vivo*, we determined their localization in hemocytes using immunofluorescence microscopy. Under an uninfected status, AaMCR showed very little co-localization with AaSR-C on the hemocyte surface (Figure 5F, the 1st row). After infection, AaSR-C co-localized well with AaMCR and DENV on the hemocyte surface (Figure 5F, the 2nd row). Moreover, knockdown of *AaMCR* eliminated AaMCR staining in hemocytes, demonstrating the specificity of the AaMCR antibody (Figure 5F, the 4th row). Because AaSR-C but not AaMCR binds DENV directly, knockdown of AaMCR did not influence DENV localization to the hemocyte membrane (Figure 5F, the 4th row). Interestingly, *AaSR-C* silencing reduced, but did not fully abolish the fluorescence staining of AaMCR, indicating that additional proteins may recruit AaMCR to hemocytes. Furthermore, we still observed DENV staining after *AaSR-C/AaMCR* silencing (Figure 5F). This is because the DENV can still bind to its cellular entry receptors [27] or some other membrane proteins on hemocytes. Without AaSR-C and AaMCR, DENV binding to hemocyte surface does not produce AMPs, but still leads to cellular entry via its receptors, resulting in DENV replication.

### AaSR-C/AaMCR induces AMPs expression to oppose DENV infection

**Figure 6:**
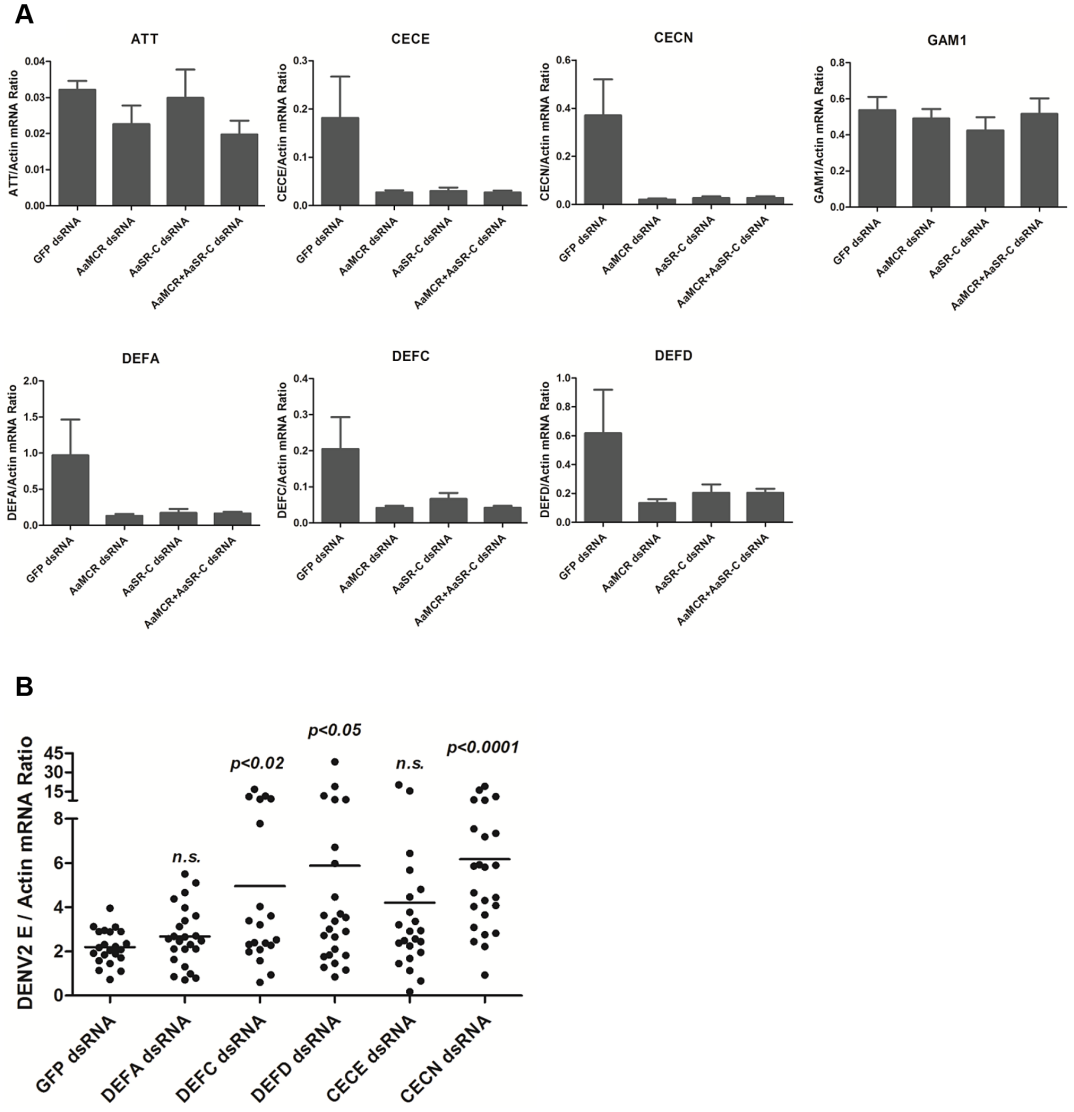
The AaMCR/AaSR-C pathway induces antimicrobial peptides production to control dengue infection. (A) Effects of AaMCR and AaSC-R knockdown on the expression of dengue-induced antimicrobial peptides. *AaSR-C* and *AaMCR* were knocked down individually or simultaneously through thoracic microinjection of dsRNA. Three days later, the gene-silenced mosquitoes were infected with (1,000 MID_50_) of DENV-2, and the *AMP* mRNA expression was assessed at 6 h post-infection via qPCR. The qPCR primers are provided in Table S2. The data is expressed as the mean± standard error of the results. The experiment was repeated 3 times. (B) Silencing of *AMPs* enhanced DENV-2 infection in *A. aegypti*. The 5 AaMCR/AaSR-C-regulated *AMPs* were knocked down through thoracic microinjection of dsRNA. DENV-2 (10 MID_50_) was inoculated at 3 days post *AMP* silencing. The viral load was assessed at 6 days post-infection via qPCR and normalized against *A. aegypti actin* (*AAEL011197*). The primers and probes used for qPCR are described in Table S2. The result was pooled from 3 independent experiments. One dot represents 1 mosquito and the horizontal line represents the mean value of the results. The data was analyzed statistically using the non-parametric *Mann-Whitney* test.

The induction of AMPs as a response to pathogen infection is a crucial mechanism of innate immunity in insects [30]. Previous studies have revealed that AMP expression is induced by DENV infection in mosquitoes, and AMPs exert antiviral activities against many types of viral infections [31], [32], [33]. We therefore examined whether the AaSR-C/AaMCR axis regulates AMP expression in response to DENV infection. Seventeen *AMP*s belonging to the *defensin* (*DEF*), *cecropin* (*CEC*), *gambicin*, and *attacin* families were identified through sequence comparison. We measured the abundance of *AMP* mRNA in DENV-2-infected and uninfected mosquitoes using qPCR after 6 h of infection. The mRNA expression of 7 *AMP*s was significantly enhanced by DENV-2 infection (more than 2 fold; Table 1). To investigate the influence of the AaSR-C/AaMCR pathway on *AMPs* regulation, we silenced *AaSR-C* and *AaMCR* individually or simultaneously through dsRNA treatment. Three days later, the gene-silenced mosquitoes were infected with DENV-2, and *AMPs* expression was assessed via qPCR at 6 h post-infection. Among the 7 DENV-induced *AMP*s, the mRNA abundance of 3 *DEF*s and 2 *CEC*s was decreased in the *AaSR-C*- and/or *AaMCR*-silenced mosquitoes compared with *GFP* dsRNA mock-treated mosquitoes, suggesting that *AaSR-C* and *AaMCR* play vital roles in DENV-mediated *AMP* induction (Figure 6A). Furthermore, double knockdown of *AaSR-C* and *AaMCR* had the same effect on *AMP* abundance as single knockdown (see Figure 6A), further indicating that AaSR-C and AaMCR function in an immune signaling pathway related to *AMPs* regulation. To elucidate the antiviral roles of these AMPs, we silenced these 5 *AMP* genes in *A. aegypti* and assessed the effect on viral burden 6 days after DENV-2 infection. The expression of these *AMP*s was significantly decreased by dsRNA treatment (Figure S6). Compared with *GFP dsRNA-*inoculated mosquitoes, mosquitoes in which *DEF C*, *DEF D*, and *CEC N* were silenced showed 2- to 3-fold enhancement of the viral burden (Figure 6B). However, knockdown of *DEF A* and *CEC E* did not significantly enhance the DENV-2 burden (see Figure 6B). Our data convincingly demonstrate that AaSR-C/AaMCR signaling induces AMPs as effectors involved in DENV limitation in *A. aegypti*.

**Table 1:** Regulation of antimicrobial peptides abundance in DENV-2 infection of *A. aegypti*.

Name	Gene Number	Function	Fold Induction (Mean)
**DEF-A**	AAEL003841	Defensin	4.06
**DEF-C**	AAEL003832	Defensin	2.4
**DEF-D**	AAEL003857	Defensin	4.68
**DEF-E**	AAEL003849	Defensin	0.97
**CEC-A**	AAEL000627	Cecropin	1.38
**CEC-B**	AAEL004223	Cecropin	1.9
**CEC-D**	AAEL000598	Cecropin	1.54
**CEC-E**	AAEL000611	Cecropin	3.23
**CEC-F**	AAEL000625	Cecropin	1.6
**CEC-G**	AAEL015515	Cecropin	1.42
**CEC-H**	AAEL017211	Cecropin	1.51
**CEC-I**	AAEL000775	Cecropin	1.25
**CEC-J**	AAEL000777	Cecropin	0.8
**CEC-N**	AAEL000621	Cecropin	3.55
**DPT-1**	AAEL004833	Diptericin	1.42
**GAM-1**	AAEL004522	Gambicin	2.88
**ATT**	AAEL003389	Attacin	3.23

## Discussion

Hosts are equipped with sophisticated machineries to detect and eliminate invading pathogens before they cause significant physiological damage. Unlike mammals, which have both innate and adaptive immune systems, insects rely solely on RNA interference and potentially on cytokine-like responses to limit viral infection [34], [35]. Despite lacking an immunoglobulin-based humoral response, insects have a functional complement-like system for warding off invading pathogens. For instance, *Anopheles* TEP1 eradicates *Plasmodium* [5], and other *Anopheles* TEPs recognize *E. coli* and *Staphylococcus aureus* to target them for phagocytosis [10]. DmMCR, which shares multiple common domains with insect TEPs and mammalian complement C3/C4/C5, has been observed to opsonize and phagocytize *C. albicans* [11]. However, whether the TEP-based complement-like system plays a role in mosquito defense against viral agents currently remains unclear.

The human complement system consists of more than 30 secreted and membrane-bound proteins. Invading pathogens are recognize by pattern receptors, including C1q, ficolin, and mannose-binding C-type lectin (MBL), subsequently triggering a complement cascade. However, the mechanism of pathogen recognition in insects may be quite different from that in mammals. Comparative genomic analyses have shown that no homologues of human C1q or ficolin exists in *A. aegypti* (Cheng G, Xiao XP, unpublished data). Moreover, the extracellular C-type lectins found in mosquitoes, which are the putative homologues of human MBL, have recently been shown to act as cellular receptors facilitating West Nile virus [26] and DENV [27] infection, rather than as antiviral pattern recognition receptors. Herein, we demonstrate that a homologue of macroglobulin complement-related factors (AaMCR) is crucial for restricting flaviviral infections. Silencing of *AaMCR* or immuno-blockade of AaMCR impaired the antiviral ability of mosquitoes. However, AaMCR does not directly interact with DENV surface proteins, suggesting that the antiviral effect of AaMCR is mediated by a certain particular adaptor. To further explore the essential players in this MCR-based antiviral response, we identified 10 proteins containing CCP domains and investigated their role in flaviviral infection in *A. aegypti*. Knockdown of *AAEL006361* enhanced flaviviral infections in the mosquitoes most significantly. Sequence analyses suggested that the protein encoded by *AAEL006361* contains 2 CCP domains at its N-terminus and is a homologue of *Drosophila* SR-C, which is a well-known pattern recognition receptor of bacterial surface components that initiates an antibacterial phagocytic response [14], [36]. In accordance with these findings, our results show that AaSR-C is capable of binding DENV surface proteins. The virion-bound AaSR-C then recruits the complement-like factor AaMCR, which induces antiviral immune factors such as AMPs to control viral infection. Indeed, in mammals, most CCP-containing complement components function as regulators of the complement cascades. For example, factor H act as a negative regulator of the alternative complement pathway [37], and C4-binding protein (C4BP) functions as an inhibitor of complement C4 [38]. This observation indicates the common distant complement system mechanism between mammals and arthropods.

TEPs generally need to be cleaved into active products following microbial attack. In human complement proteins, the alpha chain of C3 is primarily cleaved into the C3a and C3b active forms after microbial infection. C3a is involved in the inflammatory immune response, whereas C3b is further degraded into C3c and C3d. C3d plays a role in B-cell activation through binding to complement receptor 2. C3c is deposited on the pathogen surface and triggers the phagocytosis and opsonization of pathogens [29], [39]. *Anopheles* TEP1 is cleaved into 2 fragments of ∼75 kDa and ∼100 kDa in response to septic wounds and microbial infection [40]. However, emerging evidence indicates that not all iTEPs are necessarily proteolytically processed to become active. DmMCR is not cleaved in the *Drosophila* S2 supernatant, and the full-length protein takes its active form without any proteolytic processing [11]. *Ixodes ricinus* MCR lacks the lysine-arginine cleavage motif that is a typical feature of C3-complement molecules [6]. In the present study using 2 polyclonal antibodies against the N-terminus of AaMCR, we detected a ∼50-kDa band below the predicted 206-kDa full-length protein in adult female *A. aegypti* lysates through both reducing and non-reducing sodium dodecyl sulphate polyacrylamide gel electrophoresis (data not shown), indicating that AaMCR is likely constitutively cleaved into its active form for function.

Mammalian complement proteins C3 and C4, which contain TE and multiple A2M domains, are the central players in the complement system opposing pathogen invasion [41], [42]. The TE modules of C3 and C4 bind to the surface of microbes via a covalent bond, resulting in the phagocytosis and opsonization of microbes. Activated complement proteins C3 and C4 are deposited on the viral surface and subsequently eliminate virions, independent of phagocytes [41]. *Anopheles* TEP1, which has been reported to be a key factor in bacterial [10] and parasitic [5] elimination, also interacts with pathogens through its TE bond. However, not all of the TEP-pathogen interactions are apparently mediated by covalent TE bonds. *Anopheles* TEP3 (*AGAP010816*), which is an insect TEP without a TE domain [6], [43], recognizes Gram-negative bacteria for phagocytosis [10]. DmMCR, which also lacks a TE domain, interacts with *C. albicans* for pathogen eradication [11]. These observations imply an unknown alternative mechanism for the recognition of pathogens by iTEPs. Indeed, as demonstrated by our data, AaMCR, a mosquito TEP without a TE domain, recognizes DENV via AaSR-C to enhance AMP production. Given the established interaction between CCP domains and the cleaved C3 fragment or the surface components of pathogens, we speculate that the CCP domains of AaSR-C may play central role in the formation of the AaMCR/AaSR-C/virus complex.

AMPs are evolutionarily conserved components of the innate immune response. Mammalian AMPs have been demonstrated to efficiently eliminate bacteria, enveloped viruses, and fungi [44]. Given their conserved sequences, insect AMPs may play a crucial role in antiviral defense. In fact, a CEC-like peptide was recently found to be induced by DENV infection and to limit the virus in the salivary gland of *A. aegypti* [32]. Several AMPs belonging to the CEC and DEF families have been shown to present anti-DENV activity in *A. aegypti* [30]. In agreement with these findings, our results revealed that 7 mosquito *AMP*s are upregulated by DENV-2 infection, among which 3 DEFs and 2 CECs are regulated by the AaMCR-AaSR-C signalling pathway. Multiple AMPs exert antiviral activity against DENV infection. Considering the data obtained in the present study, we propose the following antiviral immune signalling pathway in *A. aegypti*: (1) viral recognition: AaSR-C recognizes the flaviviral surface protein and subsequently recruits AaMCR to form a protein-virus complex; (2) AMP induction: the conjugation of AaSR-C and AaMCR with viruses triggers an immune signalling pathway to stimulate *AMPs* expression; and (3) viral inactivation: the positive charges of AMPs can electrostatically or hydrophobically associate with the components of the viral surface [45], subsequently resulting in the flaviviral inactivation.

Similar to *Drosophila*, mosquitoes equip at least three immune signalling cascades: the Toll, Immune Deficiency (Imd), and Janus kinase (JAK)-signal transducer and activator of transcription (STAT) pathways to fight pathogenic invasion [31], [46], [47], [48]. Compared with the Imd pathway, the Toll and JAK-STAT pathways are more specific for response to viruses [31], [48]. Toll pathway is activated in response to DENV infection in *A. aegypti*, which triggers robust expression of several Toll pathway marker genes, such as *attacin*, *defensins*, and *cecropins*, to control DENV replication in mosquito tissues [49]. The JAK-STAT pathway has also been implicated in anti-viral defenses [48]. Moreover, *Dong et al.* showed that both the Toll and JAK-STAT pathways are activated by *Beauveria bassiana* infection, leading to resistance of dengue infection in *A. aegypti* [49]. Previous studies implied that both the Toll and JAK-STAT pathways may be regulated at least in part by the same signal transduction cascade. Consistent with this hypothesis, the JAK-STAT pathway can be indirectly activated by the Toll cascade in *D. melanogaster* [50]. Here, our results revealed that several mosquito AMPs, regulated by the AaMCR/AaSR-C system, are also modulated by Toll/JAK-STAT responses [49]. Given the partial overlap between Toll/JAK-STAT and AaMCR/AaSR-C regulated AMPs, we speculate that complement-like factors may directly or indirectly function together with the Toll/JAK-STAT pathways. The relationship between complement-like factors and the Toll/JAK-STAT signaling pathways remains to be elucidated in the future.

The antiviral systems of mosquitoes limit viral infection and prevent invading pathogens from causing significant physiological damage in tissues. Our results presented above show that several complement-like factors play a vital role in resistance against dengue infection in *A. aegypti*. These factors are unable to eliminate viruses from mosquitoes but can limit the viral burden to a tolerable level that does not elicit significant tissue damages in mosquitoes and that allows successful viral transmission. Without these antiviral mechanisms, viral replication could cause damages to mosquito physiology and thus decrease the mosquito life span. Previous studies have demonstrated a negative correlation between viral titers and mosquito longevity [51], [52]. We examined the life span of individual- or co-*AaMCR*/*AaSR-C* dsRNA-treated, DENV-infected mosquitoes and noted that the life span of the *AaMCR*/*AaSR-C* silenced mosquitoes was shorter (though not statistically significantly) than that of *GFP* dsRNA controls following DENV infection (Cheng G, Xiao XP, Unpublished data). Many studies suggest that arboviral infection leads to apoptosis in mosquito tissues. The midgut epithelial cells of *Culex pipiens* undergo apoptosis after West Nile virus infection [53]. Apoptosis of the salivary glands and midgut of *Aedes albopictus* was observed following Sindbis virus infection [54]. Moreover, the level of apoptosis is correlated with virus replication in infected cells [55]. We assessed apoptosis in mosquito tissues by TUNEL staining, and noted that the number of apoptotic cells in *AaMCR/AaSR-C* silenced mosquitoes was greater than in *GFP* dsRNA controls (Cheng G, Xiao XP, Unpublished data). It is plausible based on the above observations that the antiviral complement mechanism favors DENV transmission and maintenance in nature by extending the life span of DENV infected mosquitoes.

In summary, we have elucidated the antiviral activity of a system composed of the factors, involving multiple complement-related proteins in *A. aegypti*. A similar antiviral mechanism may be present in other arthropods such as *Drosophila* and *Ixodes*, which also exhibit MCR and SR-C homologues. Understanding the antiviral mechanisms of arthropods may provide novel strategies for limiting arboviral transmission in nature.

## Materials and Methods

### Ethics statement

Collection of human blood samples was conducted with approval of the local ethics committee at Tsinghua University. Human blood taken from healthy donors, who provided written informed consent, was used for mosquito blood feeding.

### Mosquitoes, cells, and viruses

*Aedes aegypti* (the Rockefeller strain) was maintained in a low-temperature illuminated incubator, model 818 (Thermo Electron Corporation, Waltham, MA) at 26°C and 80% humidity according to standard rearing procedures [31]. *Aedes albopictus* C6/36 cells were grown at 30°C in Dulbecco's modified Eagle's medium for DENVs and YFV production. *Drosophila melanogaster* S2 cells were cultured in Schneider's medium. All media were supplemented with 10% heat-inactivated foetal bovine serum, 1% L-glutamine, and 100 U/mL each of penicillin and streptomycin. Flaviviruses, including DENV-1 (Hawaii strain), DENV-2 (New Guinea C strain), DENV-3 (Guangdong strain), DENV-4 (H241 strain), and YFV (17D vaccine strain) were passaged in *A. albopictus* C6/36 cells and stocked in a −80°C ultra-freezer. The viruses for *in vivo* experiments were titrated to 50% mosquito infective dose (MID_50_). The MID_50_ measurement procedure has been described previously [26].

### Antibodies

A flavivirus E protein 4G2 monoclonal antibody was generated from a hybridoma cell line (ATCC, D1-4G2-4-15) [56]. The antibodies for tags were all purchased from Medical and Biological Laboratories Co., LTD. (Nagoya, Japan). Anti-mouse immunoglobulin G (IgG)-Alexa 488, anti-rabbit IgG-Alexa 546, and anti-human IgG-Alexa 633 were obtained from Invitrogen (Carlsbad, CA) and anti-rabbit IgG-phycoerythrin was purchased from eBioscience (San Diego, CA). The polyclonal antibodies for AaSR-C were produced in rabbits. Owing to inefficient expression of AaSR-C in *E. coli* (data not shown), we synthesised 2 peptides in the AaSR-C extracellular region (CSSGDASRRARKTGP and CNSNGTSEEPVTESD) for immunization. The polyclonal antibody was generated with 3 boosts. Murine anti-AaMCR-N polyclonal antibody was produced by the N-terminal peptide of AaMCR (AaMCR-N, 30–601 aa). *AaMCR-N* was amplified and cloned into pET28 vector for expression in *E. coli*. The cloning primers are shown in Table S2. An abundant protein expressed in inclusion bodies was washed and dissolved in 8 M urea solution and the protein was purified with a 6×His affinity column. The purified AaMCR-N protein in 2 M urea solution was inoculated into mice for polyclonal antibody production. The other AaMCR antibody (AaMCR-SP antibody), immunised with 2 synthesised peptides of the N-terminus (YNPLDPFNRQRYNP and RPRTFNPQSNRNVF), was generated in rabbits with 3 boosts.

### Bioinformatics

The sequences of TEP genes in *A. gambiae* (Ag), *A. Aegypti* (Aa), and *D. melanogaster* (Dm) were obtained from VectorBase (https://www.vectorbase.org/) and Flybase (http://flybase.org). The unrooted phylogenetic tree was built with the neighbour-joining method [57] using MEGA 5.2.2 software based on the alignment of the sequences determined using MUSCLE [58]. The bootstrap consensus tree was inferred from 500 replicates. The functional modules of MCRs, TEPs, and SR-Cs were predicted using the SMART (http://smart.embl-heidelberg.de/smart/set_mode.cgi?GENOMIC=1) and Pfam websites (http://pfam.sanger.ac.uk/). The sequence accession numbers of *iTEP* genes are *AgTEP1*, *AGAP010815*; *AgTEP2*, *AGAP008366*; *AgTEP3*, *AGAP010816*; *AgTEP4*, *AGAP010812*; *AgTEP6*, *AGAP010814*; *AgTEP9*, *AGAP010830*; *AgTEP10*, *AGAP010819*; *AgTEP13/AgMCR*, *AGAP008407*; *AgTEP15*, *AGAP008364*; *AaMCR*, *AAEL012267*; *AaTEP2*, *AAEL008607*; *AaTEP15*, *AAEL014755*; *AaTEP20*, *AAEL001794*; *AaTEP21*, *AAEL001802*; *AaTEP22*, *AAEL000087*; *AaTEP23*, *AAEL001163*; *AaTEP24*, *AAEL017023*; *DmTEP1*, *CG18096*; *DmTEP2*, *CG7052*; *DmTEP3*, *CG7068*; *DmTEP4*, *CG10363*; *DmTEP5*, *CG13079*; *DmMCR*, *CG7586. AaMCR* and *DmMCR* were named as *AaTEP13* and *DmTEP6* in the previous study [43].

### Gene silencing and viral infection in *A. aegypti*

Genes were silenced *in vivo* via dsRNA thoracic microinjection. dsRNA synthesis has been described previously [26], [59]. The primers for dsRNA synthesis are shown in Table S2. The quality of dsRNA was checked after annealing via gel electrophoresis. We have described elsewhere the detailed procedures for gene silencing and viral challenge in *A. aegypti* [26]. Briefly, female *A. aegypti* mosquitoes were cold-anaesthetised on a cold tray, and 1 μg/300 nL of dsRNA was injected into their thoraxes. The injected mosquitoes were allowed to recover 3 days under standard rearing conditions for viral infection. The mosquitoes were thoracically microinjected with 10 MID_50_/300 nL (for functional investigation) or 1,000 MID_50_/300 nL viruses (for the detection of gene expression) and then were maintained in double containers for additional investigations.

To investigate the role of AaMCR using an immuno-blocking approach, we premixed the serial diluents of AaMCR-N polyclonal antibodies with 10 MID_50_ DENV-2 and microinjected the combination into the thoraxes of mosquitoes. The treated mosquitoes were reared in double containers under standard condition. At 3 and 6 days post-infection, the inoculated mosquitoes were killed and the total RNA from the whole body was isolated to assess DENV burden with qPCR. The primers for DENV-2 detection are shown in Table S2.

### Sample preparation for immuno-blotting

Hemolymph was collected into denaturing protein loading buffer (Thermo Scientific, 26149) through proboscis clipping [60]. For the preparation of whole mosquito extracts, three mosquitoes were collected in a single tube and homogenized in 200 μl protein lysis buffer/protease inhibitor cocktail (Thermo Scientific, 1862209) using a pestle grinder system (FisherSci, 03-392-106). The lysates was centrifuged to remove debris and lipids. Hemolymph and whole mosquito extracts were denatured in protein loading buffer at 65°C for 5 min for SDS-PAGE and immuno-blotting [40], [60].

### Detection of viral burden in mosquitoes by qPCR

The whole body of infected mosquitoes was homogenised in Buffer I of RNA isolation kit (Qiagen, 74106) with a Pestle Grinder System (FisherSci, 03-392-106). The detailed procedure of total RNA isolation is described in the kit manual. Complementary DNA (cDNA) was randomly reverse-transcribed using a cDNA Synthesis Kit (Bio-Rad, 1708891). The viral burden was then quantified with qPCR. The primers and probes for *DENV 1-4 E* and *YFV E* genes are shown in Table S2. The amount of virus was normalised with *A. aegypti actin* (*AAEL011197*).

### Detection of the *AMP* abundance in *A. aegypti*

We measured AMP abundance in DENV-2 infected or mock mosquitoes using qPCR after 6 h of infection. DENV-2 virus (1,000 MID_50_) was thoracically inoculated into mosquitoes. The mock mosquitoes were microinjected with phosphate-buffered saline (PBS). Six hours post-viral inoculation, the whole bodies of mosquitoes were homogenised for total RNA isolation. cDNA was randomly reverse-transcribed with a cDNA Synthesis Kit. The abundance of *AMP* genes was quantified with qPCR. The primers for *AMPs* detection are shown in Table S2. The number of genes was normalized with *A. aegypti actin*.

### Protein generation in a *Drosophila* expression system

The genes of *AaMCR* and *AaSR-C-Ex* were amplified from a cDNA library of adult female *A. aegypti*. The PCR fragments were inserted into the pMT/BiP/V5-His A vector (Invitrogen, V4130-20), and the recombinant plasmids were transfected into *Drosophila* S2 cells in combination with the hygromycin selection vector pCo-Hygro for stable cell construction. The primers for PCR and gene cloning are shown in Table S2. The transfected cells were selected using 300 μg/mL hygromycin-B (Invitrogen, 10687-010) for 4 weeks. The resistant cells were grown in spinner flasks, switched to serum-free medium (GIBCO, Invitrogen, 10486025) for 3 days, and induced with copper sulphate at a final concentration of 500 μM for 4 days. The culture medium was cleared with centrifugation at 1,000 × *g* for 5 min and collected for protein purification with a metal affinity resin (Clontech, Mountain View, CA, PT1320-1). The protein was eluted with 150 mM imidazole, extensively dialysed against PBS (pH 7.8), and concentrated via centrifugal filtration through a 5-kDa filter (Millipore Corp., Bedford, MA, pLCC07610) [26]. The protein concentration was measured using Protein Assay Dye (Bio-Rad, 500-0006) and a Nanodrop 2000c spectrophotometer. The protein purity was checked with sodium dodecyl sulphate polyacrylamide gel electrophoresis, and the specificity of purification was confirmed with immunoblotting using mouse mAbs (Medical and Biological Laboratories Co., LTD.).

### Co-immunoprecipitation

To perform the co-IP assays with purified proteins (see Figures 4D, 5C, and S4B), DENV-2 E, AaMCR-a, or AaSR-C was expressed and purified using a *Drosophila* expression system. Two micrograms each of the purified proteins was incubated at 4°C for 2 hr. Subsequently, 1 μg of a baited antibody was added, and a 2 hr incubation was performed to pull down the protein complex. For experiments involving cell supernatants (see Figure 5A), recombinant plasmids encoding AaMCR fragments were transiently transfected into *Drosophila* S2 cells using a transfection kit (Qiagen, 301425). Following copper induction, the cell supernatant was collected 48 hr after transfection, and AaMCR expression was detected via western blotting using an anti-HA antibody. The supernatant was incubated with 5 μg of purified AaSR-C-Ex protein for the co-IP assay. The experimental details of the co-IP assays are described in the Classic IP kit manual (Thermo Scientific, 26146).

### ELISA

A microtiter test plate (Nunc, Roskilde, Denmark) was coated with 2 μg of bait protein overnight at 4°C. After 5 washes with PBS containing 0.05% Tween 20 (PBST), 5 μg of purified protein or cell lysate was added to each well, followed by incubation at room temperature (RT) for 2 hr. The wells were then washed 5 times with PBST. after which the primary antibody was added, and incubation was continued at RT for 2 hr. The wells were washed again, and 100 μL of a secondary IgG-horseradish peroxidase antibody was added. Following incubation at RT for 1 hr, a commercial peroxidase substrate system was applied (Kirkegaard & Perry Laboratories, Inc., MA, 50-76-11), and the optical density at 450 nm was measured with an ELISA reader.

The interaction between the proteins and DENV-2 virions was also measured via ELISA. In this procedure, the microtiter test plate was coated with 2 μg of recombinant protein at 4°C overnight. After 5 washes with PBST, 2 μg of purified inactivated DENV-2 virions (MicroBix, Canada, EL-22-02-001) in PBS was added to each well, followed by incubation for 2 hr at 4°C. After washing with PBST, a flavivirus E protein 4G2 mAb was added, followed by incubation for an additional 2 hr at 4°C. The analysis followed the procedure outlined above.

### Flow cytometry

The full-length *AaSR-C* was cloned into pMT/BiP/V5-His A for the stable cell construction. An Myc tag was introduced into the N-terminal of AaSR-C protein. AaSR-C expression on the cell surface was induced with copper sulphate at a final concentration of 500 μM. Inactivated DENV-2 virions were incubated with AaSR-C-expressing cells at 4°C for 2 hr, and the non-induced cells or mock cells with DENV-2 virions served as control groups. Cells were then fixed with 4% paraformaldehyde (USB Corporation, Cleveland, OH) and stained with primary and second antibodies. The treated cells were then examined using a FACS Calibur flow cytometer (BD Biosciences, San Diego, CA). Dead cells were excluded on the basis of forward and side light scatter. Data was analysed using FlowJo software.

### Immunofluorescence microscopy

Hemocytes were isolated and placed on sialylated slides (PGC Scientific, Gaithersburg, MD), washed in PBS, and fixed in 4% paraformaldehyde at 4°C for 2 h. Detailed mosquito dissection procedures have been described previously [61]. Hemolymph was dried on the sialylated slides. Samples were blocked in PBS with 1% bovine serum albumin and 0.1% Triton X-100 at RT for 2 hr before antibody incubation. After being incubated with primary and secondary antibodies, the slides were imaged with the multitrack mode of Zeiss LSM 780 meta confocal microscope.

### Plaque assay

The numbers of DENV and YFV virions were measured in plaque assay. Briefly, mosquitoes were sacrificed on serial days post-infection, and individual mosquitoes were then homogenized in 200 μl PBS using a pestle grinder system (FisherSci, 03-392-106). The samples were centrifuged for 10 min at 4°C and the supernatant were collected for virological assays. Vero cells were seeded into flat-bottomed 12-well plates and grown overnight. The mosquito lysates were serially diluted 10 times (10^−1^∼10^−6^) with DMEM and each dilution being included in 3 parallel wells. After 2 hrs at 37°C, the fresh medium was replaced in each well, and the cells were covered with melting Agarose (Lonza, 50302) to be culture for additional days. The cells were stained with 1% crystal violet. The viral titration was calculated based on the number of plaque-forming units.

## Supporting Information

Figure S1 **Detection of the number of infectious virions in** ***AaMCR*** **-silenced mosquitoes, immunostaining of native AaMCR protein and regulation of** ***AaMCR*** **abundance in DENV infected mosquito tissues.** (A-E) Silencing *AaMCR* enhanced the number of DENVs and YFV virions in *A. aegypti*. 10 MID_50_ DENVs or YFV were respectively inoculated at 3 days post *AaMCR* silencing. The infected mosquitoes were collected to detect viruses loads by plaque assay. No less than 8 mosquitoes were detected in one group. Data is expressed as the mean ± standard error. The data was statistically analyzed by non-parametric *Mann-Whitney* test. (F) Purification of N-terminal peptide of AaMCR peptide (AaMCR-N, 30–601 aa) in *E. coli*. The *AaMCR-N* fragment (30–601 aa) was cloned into pET28 DNA vector and expressed in *E. coli* BL21 DE3 strain. The recombinant protein, which was expressed in inclusion body, was dissolved in 8M Urea and purified by Ni-His column for antibody generation. (G) Validation of AaMCR-N polyclonal antibody. A mouse-derived AaMCR-N polyclonal antibody, generated by a recombinant peptide from *E. coli* (F), was used to probe native AaMCR in mosquito lysates or S2-expressed AaMCR-N recombinant peptide. The same samples probed by mice pre-immune serum served as a negative control. (H) Detection of native AaMCR in mosquito hemolymph. Pure hemolymph was then collected by proboscis clipping. The same samples probed by mice pre-immune serum served as a negative control. (I) Confirmation of native band by AaMCR-SP antibody. The two peptides of N-terminal AaMCR-N were synthesized for immunization in rabbit. The polyclonal antibody, designated as AaMCR-Synthesized Peptides antibody (AaMCR-SP antibody), was used to detect native AaMCR in mosquito lysates. The same samples detected by rabbit pre-immune serum was used as a negative control. (J) Schematic representation of the different AaMCR fragments mapped onto the whole protein. The functional modules were predicted in SMART (http://smart.embl-heidelberg.de/smart/set_mode.cgi?GENOMIC=1) and Pfam websites (http://pfam.sanger.ac.uk/). (K) *AaMCR* regulation by DENV-2 infection. DENV-2 (1,000 MID_50_) or PBS was microinjected into mosquitoes. Total RNA was isolated from whole mosquitoes (i), salivary glands (ii), hemolymph (iii) and midgut (iv) to determine *AaMCR* expression by qPCR. The qPCR primers of *AaMCR* were described in Table S2. Data was represented as the mean ± standard error.
(PDF)

Figure S2 ***AAEL006361*** **dsRNA-mediated gene silencing enhanced dengue infections in** ***A. aegypti*** **.** (A-B) dsRNA-mediated *AAEL006361* silencing. The mosquitoes were microinjected with 1 ug *AAEL006361* or *GFP* dsRNA respectively, and then sacrificed to assess the effect by qPCR at 3 and 6 days (A) and by Western-Blotting (B) at 6 days post dsRNA treatment. The primers of dsRNA synthesis and qPCR were described in Table S2. (C-D) Silencing *AAEL006361* enhanced DENV infections of *A. aegypti*. 10 MID_50_ DENV-1 (C) or DENV-3 (D) was inoculated at 3 days after dsRNA microinjection. The effect of virus burden was measured at 6 days post infection by qPCR and normalized by *A. aegypti actin*. The experiment was repeated 3 times.
(PDF)

Figure S3 **Validation of AaSR-C polyclonal antibody and the regulation of** ***AaSR-C*** **abundance in DENV-2 infection.** (A) Detection of native AaSR-C in mosquito lysates. AaSR-C polyclonal antibody was generated in rabbit by synthesized peptides in AaSR-C extracellular region. AaSR-C native protein detected by the antibody was showed in the left panel; the detection of preimmune serum served as a negative control (Right panel). (B) The regulation of *AaSR-C* abundance in DENV-2 infection. DENV-2 (1,000 MID_50_) or PBS was microinjected into *A. aegypti*. Total RNA was isolated from whole mosquitoes (i), salivary glands (ii), hemolymph (iii) and midgut (iv) at a time course to determine *AaSR-C* expression by qPCR. The qPCR primers of *AaSR-C* were described in Table S2. Data was represented as the mean ± standard error.c
(PDF)

Figure S4 **The interaction between AaMCR-a and AaSR-C-Ex purified proteins.** (A) AaMCR-a interacts with AaSR-C-Ex by ELISA assay. AaSR-C or BSA purified protein was coated at 4°C overnight on each plate well. Subsequently, AaMCR purified protein was added into the wells to determine the interaction. A mouse anti-HA antibody was used as the detecting antibody. Data was expressed as the mean ± standard error. The experiment was reproduced 3 times. (B) AaMCR-a binds to AaSR-C-Ex by co-IP. 2 ug each of AaMCR-a and AaSR-C-Ex purified proteins was premixed at 4°C for 2 hrs. The complex was pulled down by a rabbit anti-V5 and probed with a mouse anti-HA antibody. The experiment was repeated 3 times with the similar result.
(PDF)

Figure S5 **Detection of DENV spread in various mosquito tissues through the infection of oral feeding.** (A-C) DENV spread in various mosquito tissues through the infection of oral feeding. We silenced *AaMCR*, *AaSR-C* and both of them with dsRNA via intra-thoracic microinjection. Three days after dsRNA treatment, the mosquitoes were fed with Vero cells-generated DENV-2 and fresh human blood. The specific tissues were dissected at 3 days (A), 6 days (B), 9 days (C) to evaluate the kinetics of viral dissemination by qPCR. Data was expressed as the mean ± standard error. Three samples of a tissue were pooled to isolate total RNA. No less than 9 samples were measured in one group. (D) Viral number in salivary glands extraction (SGE). *AaMCR*, *AaSR-C* and both of them were silenced by dsRNA via intra-thoracic microinjection in *A.aegypti*. *GFP* dsRNA inoculation served as a negative control. Nine days post DENV-2 infection, the salivary glands were dissected and grinded in PBS buffer. The DENV number in per SGE was measured by plaque assay. No less than 6 samples were detected in one group. (A-D) The data was statistically analyzed by non-parametric *Mann-Whitney* test.
(PDF)

Figure S6 **Silencing efficiency of** ***AMP*** **genes in** ***A. aegypti.*** (A-E) *AMP* genes were silenced in mosquitoes respectively. *GFP* dsRNA served as a mock control. The mosquitoes were sacrificed at 9 days after dsRNA inoculation. The expression of *AMP* genes was determined by qPCR and normalized by *A. aegypti actin*. The qPCR primers were shown in Table S2. The non-parametric *Mann-Whitney* test was used for statistical analysis.
(PDF)

Table S1 **The characterization of proteins containing CCP domain in** ***A. aegypti.***
(PDF)

Table S2 **Primers and probes for qPCR, dsRNA synthesis and genes cloning.**
(PDF)
