# Supplementary figures and images for: Complement-Related Proteins Control the Flavivirus Infection of Aedes aegypti by Inducing Antimicrobial Peptides
Source: PLoS Pathog. 2014 Apr 10;10(4):e1004027. doi: 10.1371/journal.ppat.1004027 (PMC3983052; doi:10.1371/journal.ppat.1004027)

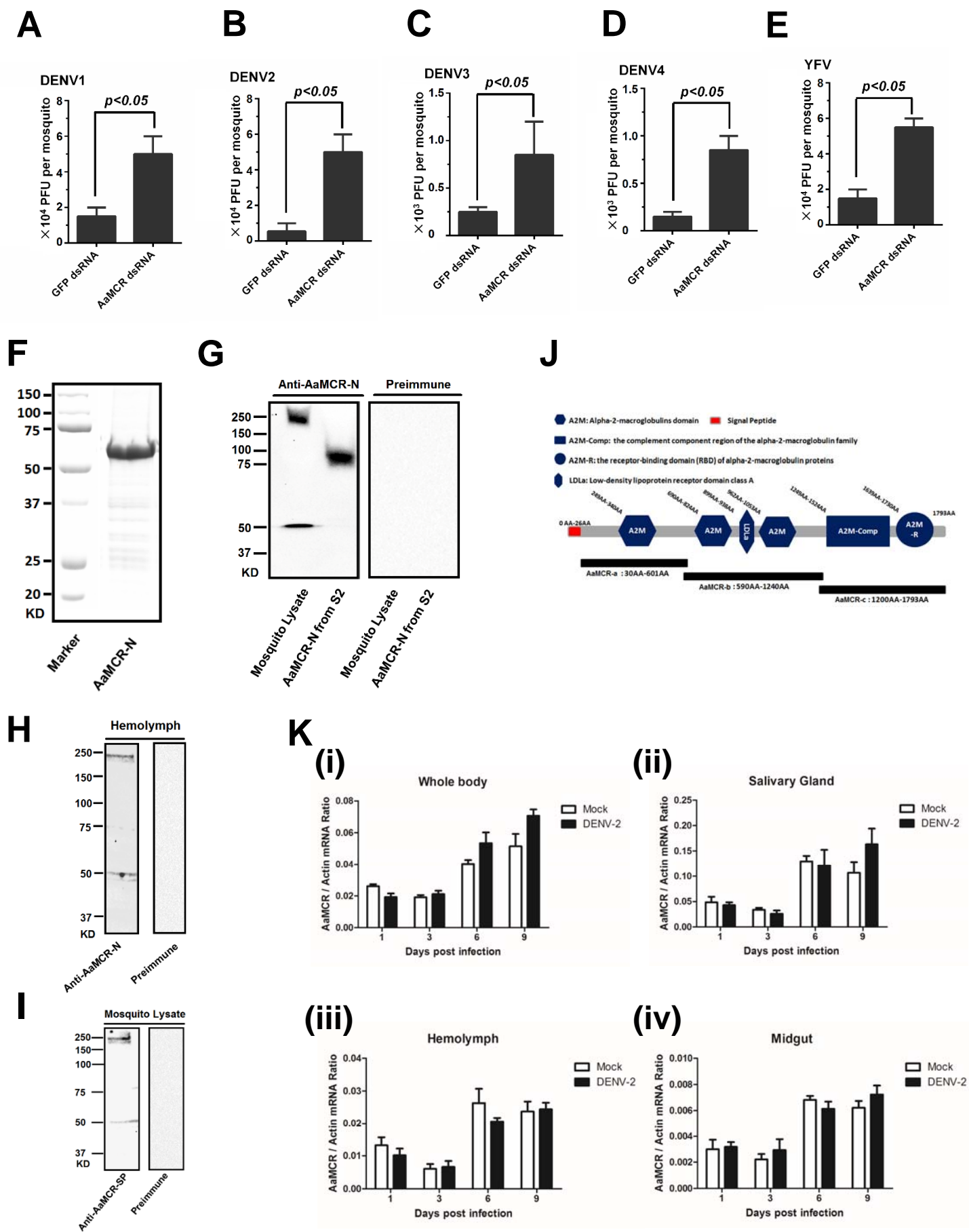

Supplement: Figure S1 — Detection of the number of infectious virions in AaMCR -silenced mosquitoes, immunostaining of native AaMCR protein and regulation of AaMCR abundance in DENV infected mosquito tissues. (A-E) Silencing AaMCR enhanced the number of DENVs and YFV virions in A. aegypti. 10 MID50 DENVs or YFV were respectively inoculated at 3 days post AaMCR silencing. The infected mosquitoes were collected to detect viruses loads by plaque assay. No less than 8 mosquitoes were detected in one group. Data is expressed as the mean ± standard error. The data was statistically analyzed by non-parametric Mann-Whitney test. (F) Purification of N-terminal peptide of AaMCR peptide (AaMCR-N, 30–601 aa) in E. coli. The AaMCR-N fragment (30–601 aa) was cloned into pET28 DNA vector and expressed in E. coli BL21 DE3 strain. The recombinant protein, which was expressed in inclusion body, was dissolved in 8M Urea and purified by Ni-His column for antibody generation. (G) Validation of AaMCR-N polyclonal antibody. A mouse-derived AaMCR-N polyclonal antibody, generated by a recombinant peptide from E. coli (F), was used to probe native AaMCR in mosquito lysates or S2-expressed AaMCR-N recombinant peptide. The same samples probed by mice pre-immune serum served as a negative control. (H) Detection of native AaMCR in mosquito hemolymph. Pure hemolymph was then collected by proboscis clipping. The same samples probed by mice pre-immune serum served as a negative control. (I) Confirmation of native band by AaMCR-SP antibody. The two peptides of N-terminal AaMCR-N were synthesized for immunization in rabbit. The polyclonal antibody, designated as AaMCR-Synthesized Peptides antibody (AaMCR-SP antibody), was used to detect native AaMCR in mosquito lysates. The same samples detected by rabbit pre-immune serum was used as a negative control. (J) Schematic representation of the different AaMCR fragments mapped onto the whole protein. The functional modules were predicted in SMART (http://smart.embl-heidelberg.de/ [file ppat.1004027.s001.pdf]

**A**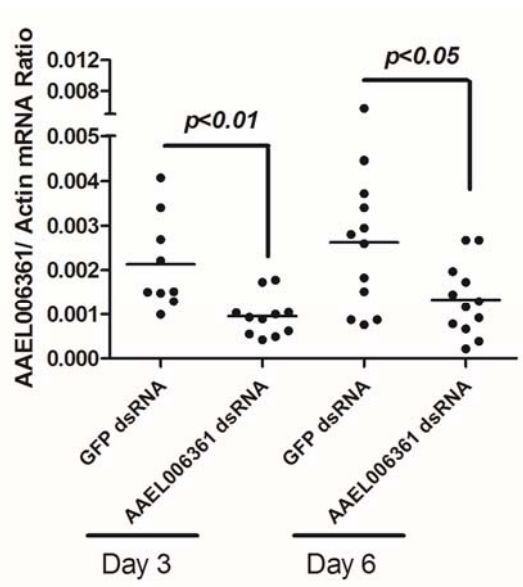**B**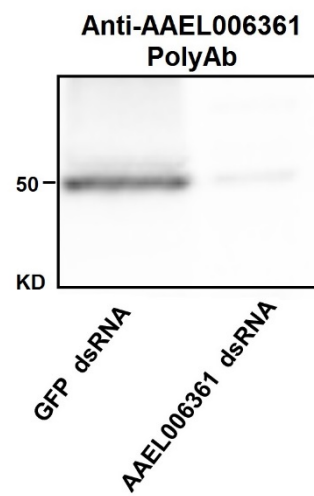**C**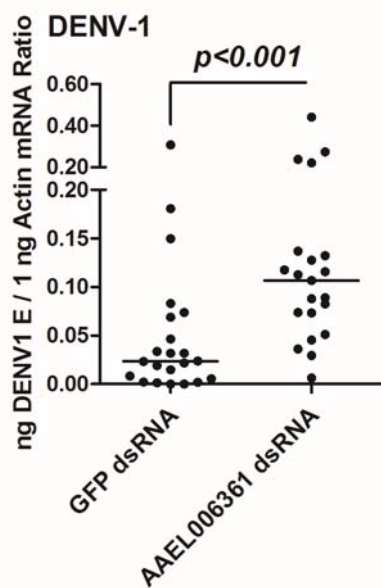**D**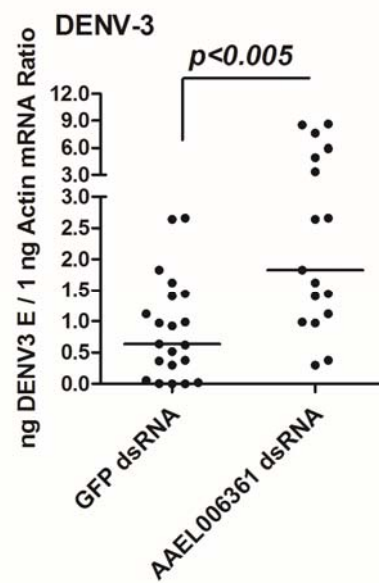

Supplement: Figure S2 — AAEL006361 dsRNA-mediated gene silencing enhanced dengue infections in A. aegypti . (A-B) dsRNA-mediated AAEL006361 silencing. The mosquitoes were microinjected with 1 ug AAEL006361 or GFP dsRNA respectively, and then sacrificed to assess the effect by qPCR at 3 and 6 days (A) and by Western-Blotting (B) at 6 days post dsRNA treatment. The primers of dsRNA synthesis and qPCR were described in Table S2. (C-D) Silencing AAEL006361 enhanced DENV infections of A. aegypti. 10 MID50 DENV-1 (C) or DENV-3 (D) was inoculated at 3 days after dsRNA microinjection. The effect of virus burden was measured at 6 days post infection by qPCR and normalized by A. aegypti actin. The experiment was repeated 3 times. (PDF) [file ppat.1004027.s002.pdf]

**A**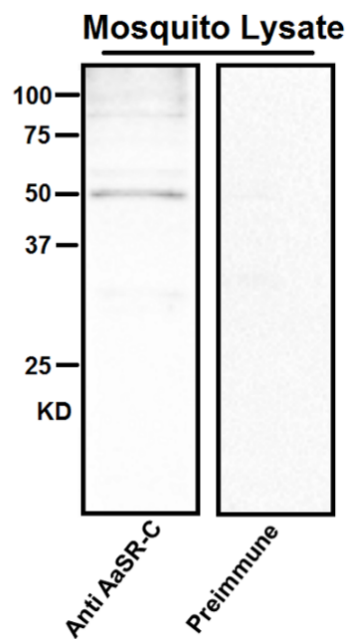**B****(i)**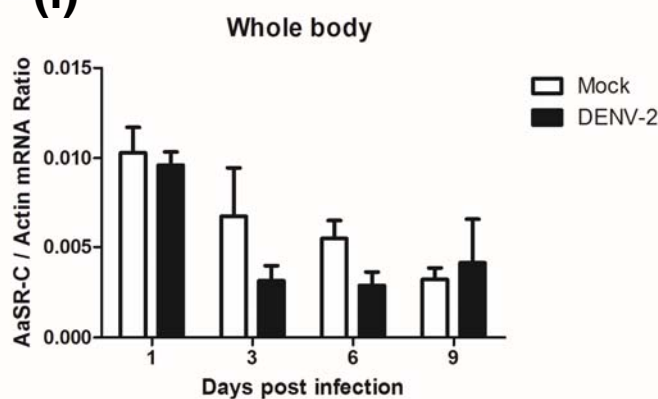**(ii)**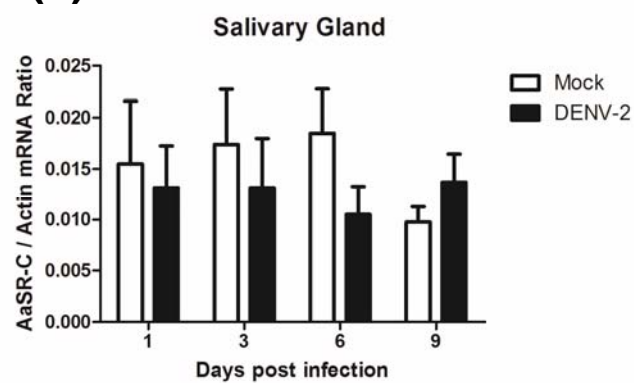**(iii)**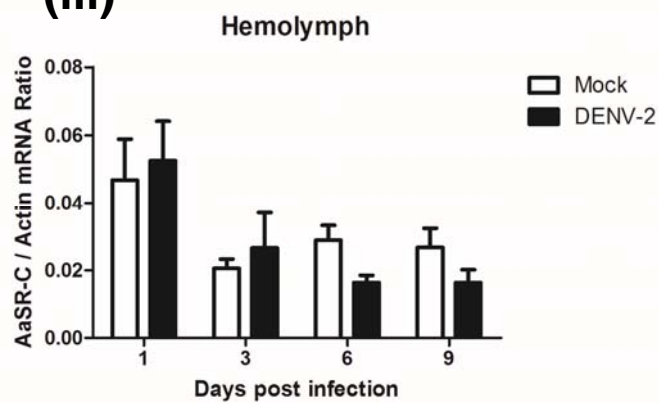**(iv)**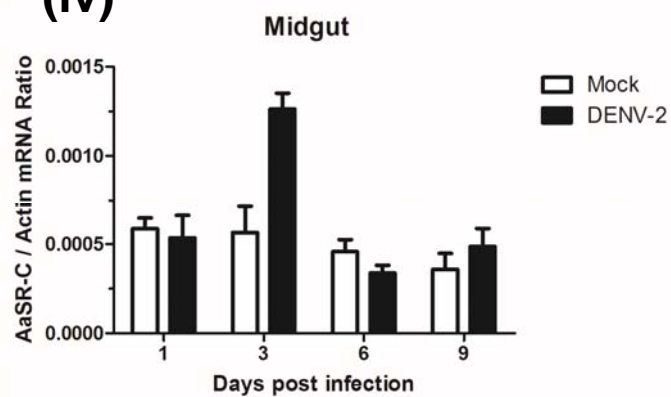

Supplement: Figure S3 — Validation of AaSR-C polyclonal antibody and the regulation of AaSR-C abundance in DENV-2 infection. (A) Detection of native AaSR-C in mosquito lysates. AaSR-C polyclonal antibody was generated in rabbit by synthesized peptides in AaSR-C extracellular region. AaSR-C native protein detected by the antibody was showed in the left panel; the detection of preimmune serum served as a negative control (Right panel). (B) The regulation of AaSR-C abundance in DENV-2 infection. DENV-2 (1,000 MID50) or PBS was microinjected into A. aegypti. Total RNA was isolated from whole mosquitoes (i), salivary glands (ii), hemolymph (iii) and midgut (iv) at a time course to determine AaSR-C expression by qPCR. The qPCR primers of AaSR-C were described in Table S2. Data was represented as the mean ± standard error.c (PDF) [file ppat.1004027.s003.pdf]

**A**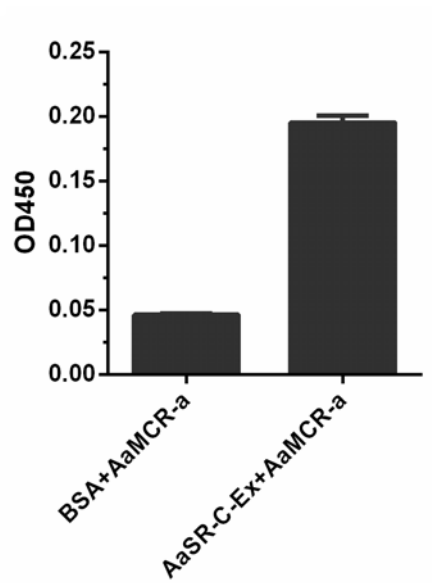**B**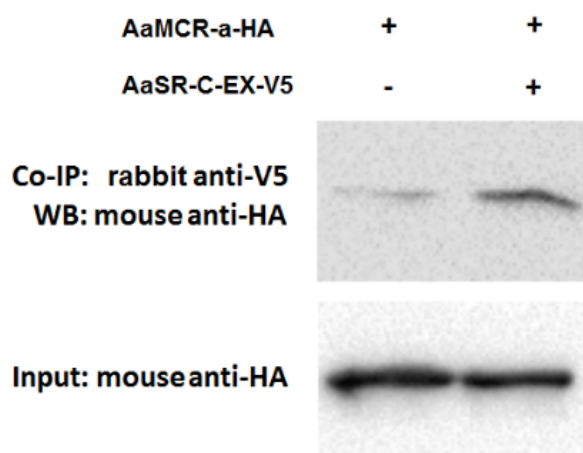

Supplement: Figure S4 — The interaction between AaMCR-a and AaSR-C-Ex purified proteins. (A) AaMCR-a interacts with AaSR-C-Ex by ELISA assay. AaSR-C or BSA purified protein was coated at 4°C overnight on each plate well. Subsequently, AaMCR purified protein was added into the wells to determine the interaction. A mouse anti-HA antibody was used as the detecting antibody. Data was expressed as the mean ± standard error. The experiment was reproduced 3 times. (B) AaMCR-a binds to AaSR-C-Ex by co-IP. 2 ug each of AaMCR-a and AaSR-C-Ex purified proteins was premixed at 4°C for 2 hrs. The complex was pulled down by a rabbit anti-V5 and probed with a mouse anti-HA antibody. The experiment was repeated 3 times with the similar result. (PDF) [file ppat.1004027.s004.pdf]

**A**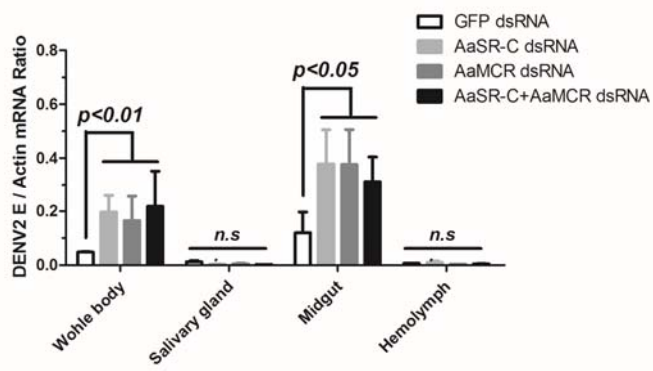**B**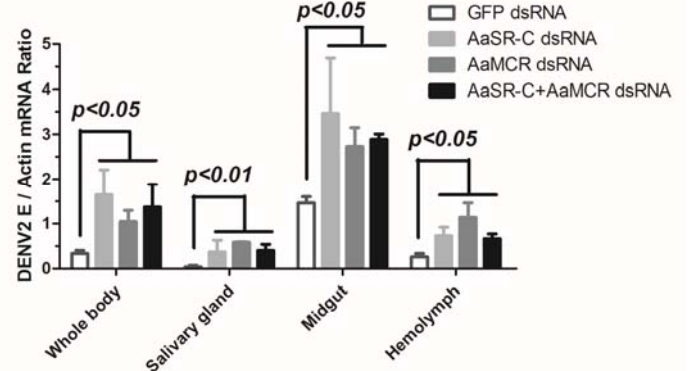**C**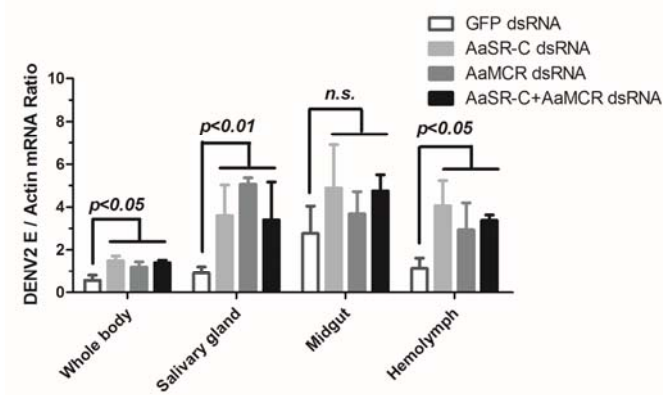**D**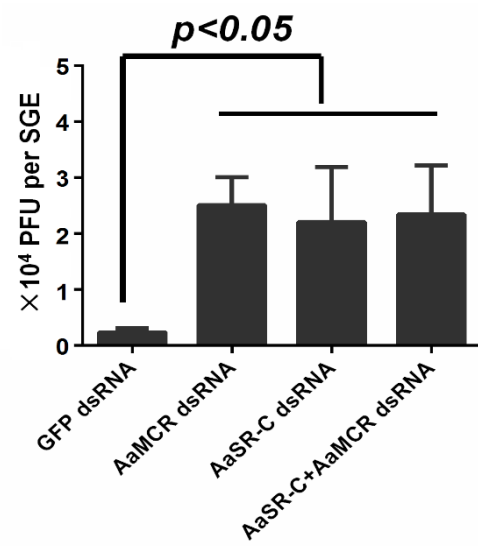

Supplement: Figure S5 — Detection of DENV spread in various mosquito tissues through the infection of oral feeding. (A-C) DENV spread in various mosquito tissues through the infection of oral feeding. We silenced AaMCR, AaSR-C and both of them with dsRNA via intra-thoracic microinjection. Three days after dsRNA treatment, the mosquitoes were fed with Vero cells-generated DENV-2 and fresh human blood. The specific tissues were dissected at 3 days (A), 6 days (B), 9 days (C) to evaluate the kinetics of viral dissemination by qPCR. Data was expressed as the mean ± standard error. Three samples of a tissue were pooled to isolate total RNA. No less than 9 samples were measured in one group. (D) Viral number in salivary glands extraction (SGE). AaMCR, AaSR-C and both of them were silenced by dsRNA via intra-thoracic microinjection in A.aegypti. GFP dsRNA inoculation served as a negative control. Nine days post DENV-2 infection, the salivary glands were dissected and grinded in PBS buffer. The DENV number in per SGE was measured by plaque assay. No less than 6 samples were detected in one group. (A-D) The data was statistically analyzed by non-parametric Mann-Whitney test. (PDF) [file ppat.1004027.s005.pdf]

**A**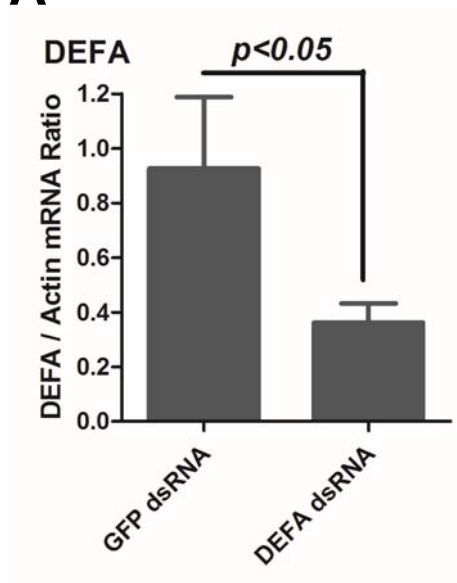**B**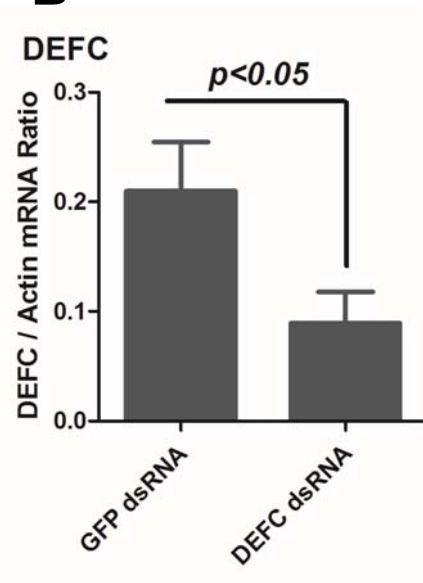**C**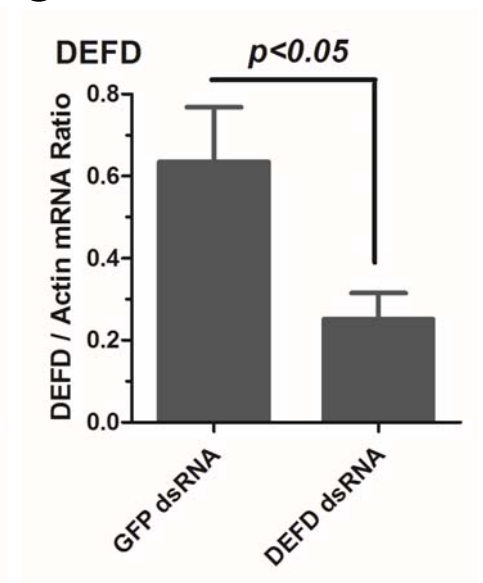**D**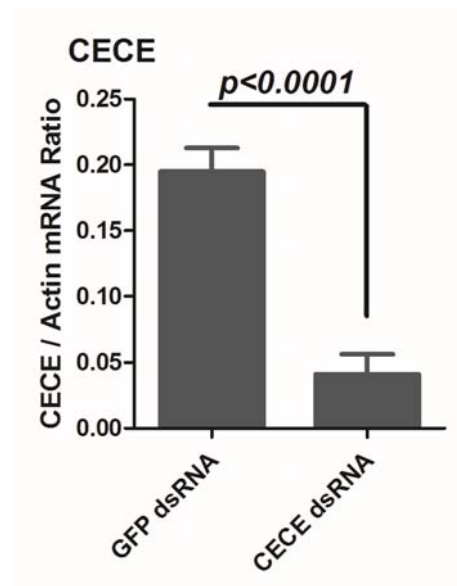**E**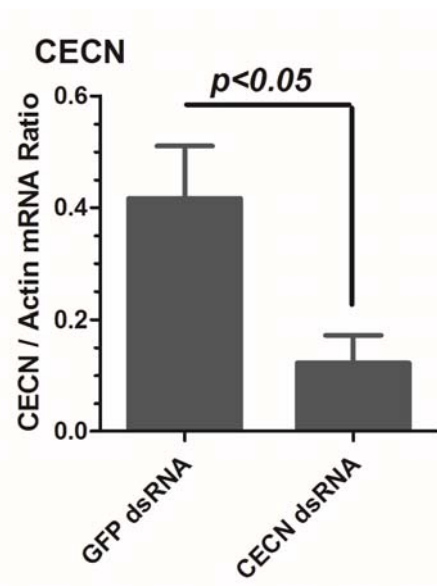

Supplement: Figure S6 — Silencing efficiency of AMP genes in A. aegypti. (A-E) AMP genes were silenced in mosquitoes respectively. GFP dsRNA served as a mock control. The mosquitoes were sacrificed at 9 days after dsRNA inoculation. The expression of AMP genes was determined by qPCR and normalized by A. aegypti actin. The qPCR primers were shown in Table S2. The non-parametric Mann-Whitney test was used for statistical analysis. (PDF) [file ppat.1004027.s006.pdf]
