# Supplementary material for: Complement-Related Proteins Control the Flavivirus Infection of Aedes aegypti by Inducing Antimicrobial Peptides
Source: PLoS Pathog. 2014 Apr 10;10(4):e1004027. doi: 10.1371/journal.ppat.1004027 (PMC3983052; doi:10.1371/journal.ppat.1004027)
Supplement: Table S1 — The characterization of proteins containing CCP domain in A. aegypti. (PDF) [file ppat.1004027.s007.pdf]

Table S1. The characterization of proteins containing CCP domain in *A. aegypti*

Gene number	Number of CCP domains	Trans-membrane	Signal peptide	Length(bp)
**AAEL004725**	5	No	No	2646
**AAEL005432**	1	Yes	Yes	4284
**AAEL005982**	3	No	No	4674
**AAEL006355**	2	Yes	No	1929
**AAEL006361**	2	Yes	Yes	1485
**AAEL008069**	10	Yes	No	10203
**AAEL008929**	11	No	No	3060
**AAEL009266**	17	No	Yes	5001
**AAEL012452**	4	No	No	3879
**AAEL014356**	3	Yes	No	2640
