# Supplementary material for: Complement-Related Proteins Control the Flavivirus Infection of Aedes aegypti by Inducing Antimicrobial Peptides
Source: PLoS Pathog. 2014 Apr 10;10(4):e1004027. doi: 10.1371/journal.ppat.1004027 (PMC3983052; doi:10.1371/journal.ppat.1004027)
Supplement: Table S2 — Primers and probes for qPCR, dsRNA synthesis and genes cloning. (PDF) [file ppat.1004027.s008.pdf]

Table S2. Primers and probes for qPCR, dsRNA synthesis and genes cloning

**Primers for cloning into pAc5.1/V5-His/A**	**Upper primer**	**Lower primer**	
AaSR-C-Ex (myc tag in the N-terminal,V5 tag in the C-terminal)	AGAGCGGCCGCGCCACCATGGAACAAAAACTCATCTCAGAAGAGGATCTG<br>AATATGCATACCGGT GTTTTGGCGTTGGTTGTGG	GGACTCGAGTTCGTAACCGTCCGCGGC	
AaMCR-a (HA tag in C-terminal)	AGAGGTACCGCCACCATGAATCCCAACGACGTGCCA	AGACTCGAGTCAAGCGTAATCTGGAACATCGTATGGGTACTCCAT<br>CCGACCTGGATATA	
AaMCR-b (HA tag in C-terminal)	AGAGGTACCGCCACCATGCCAATCGATCCGCTATAT	AGACTCGAGTCAAGCGTAATCTGGAACATCGTATGGGTACTCCGT<br>TTTCTTGTTACGTTGT	
AaMCR-c (HA tag in C-terminal)	AGAGAATTCGCCACCATGGCAACATCTCTACTATCGT	AGACTCGAGTCAAGCGTAATCTGGAACATCGTATGGGTAATCGTT<br>CCAAAAGATCCAA	
**Primers for cloning into pMT/BiP/V5-His/A**	**Upper primer**	**Lower primer**	
DENV-2 Envelop gene (3×FLAG tag in N-terminal)	CGAAGATCTGCCACCATGGACTACAAAGACCATGACGGTGATTATAAAGAT<br>CATGACATCGATTACAAGGATGACGATGACAAGATGCGTTGCATAGGAATA	AGACTCGAGTCAATGGTGATGGTGATGATGTTGGCCGATAGAACT<br>TC	
AaSR-C-Ex (V5 tag in C-terminal)	CAACAGATCTTTGGTTGTGGATATTTCCGCTTA	GGACTCGAGTTCGTAACCGTCCGCGGC	
AaSR-C-Full (V5 tag in C-terminal / Myc in N-terminal)	CAACAGATCTGCCACCATGGAACAAAAACTCATCTCAGAAGAGGATCTGA<br>ATATGCATACCGGTAACATCGATCGCTTATCGC	GGACTCGAGTGGTAACCGCATACTAGCAACAC	
AaMCR-a (3XHA tag in C-terminal))	CGAAGATCTGCCACCATGTACCCATACGATGTTCCTGACTATGCGGGCTAT<br>CCCTATGACGTCCCGGACTATGCAGGATCCTATCCATATGACGTTCCAGAT<br>TACGCTAATCCCAACGACGTGCCA	CAGACTCGAGTCAATGGTGATGGTGATGATGACCGGTACGCTCCA<br>TCCGACCTGGATATA	
**The primers for genes cloning(pET28)**	**Upper primer**	**Lower primer**	
AaMCR--N	TATCTAGCTAGCAATCCCAACGACGTGCC	TTATCCCTCGAGCTCCATCCGACCTG	
**The primers for RT-QPCR**	**Upper primer**	**Lower primer**	**Probe (for Taqman QPCR)**
AaMCR	TCCGTCCCGGTCAGATTTACAA	TCTACGCCGTCACGGGAGATAC	
AAEL004725	AAGGGAAGTGGAAGCGACAG	AGGTTTGGCGACCTCTGAAG	
AAEL005432	GGCGTGGGGAAAGCGTTATT	TTCGGGGGTAAGAATTGTGACG	
AAEL005982	ATGCGGTCGGCTCGTTTGA	CCCTCCGTCCCATCCTTACAC	
AAEL006355	AGGCGCCGGATCTCTGTGG	CTTCAACGCTTTCGGGTGGAC	
AAEL006361	AGCGGATACAAGTTGGCTGGAT	TCCGCATAGATCGCTCACTTCA	
AAEL008069	CATGCGTTCCGATGATGTGTCC	TCGCCAAAGTGGTGGTCTACCT	
AAEL008929	GAGGACGCAGCTGGTGTGGA	GCTCGCCGTTGTAGTTGTTCG	
AAEL009266	TCAACGGCAGCAGCTCTTACAC	GGCCCATTCTCCATTCCAACA	
AAEL012452	AACACCCCCGCACCTTATGTG	AAAGTTGTCACCCCCATTGTCG	
AAEL014356	GAATGCCCGCCTCAATCTGG	GGTGCCGGTAGTGCTGGTTC	
DENV-1 Envelop gene	GACACCACACCCTTTGGACAA	CACCTGGCTGTCACCTCCAT	FAM-AGAGGGTGTTTAAAGAGAAAGTTGACACGCG-TAMRA
DENV-2 Envelop gene	CATTCCAAGTGAGAATCTCTTTGTCA	CAGATCTCTGATGAATAACCAACG	FAM-ATGCTGAAACGCGAGAGAAACCGC-TAMRA
DENV-3 Envelop gene	GGGAAAACCGTCTATCAATA	CGCCATAACCAATTTCATTGG	FAM-CACAGTTGGCGAAGAGATTCTCAAGAGGA-TAMRA
DENV-4 Envelop gene	TGAAGAGATTCTCAACCGGAC	AATCCCTGCTGTTGGTGGG	FAM-TCATCACGTTTTTGCGAGTCCTTTCCA-TAMRA
YFV Envelop gene	TGGCATATTCCAGTCAACCTTCT	GAAGCCCAAGATGGAATCAACT	FAM-TTCCACACAATGTGGCATG–MGB
A.Aegypti Actin	GAACACCCAGTCCTGCTGACA	TGCGTCATCTTCTCACGGTTAG	FAM-AGGCCCCGCTCAACCCGAAG-TRAMA
DEFA	CTATCAGGCTGCCGTGGAG	CAATGAGCAGCACAAGCACTATC	
DEFC	CTTTGTTTGATGAACTTCCGGAG	GAACCCACTCAGCAGATCGC	
DEFD	GGCGTTGGTGATAGTGCTTG	CACACCTTCTTGGAGTTGCAG	
DEFE	GTGCGGGACACTGTCTAGCC	CAATCCTAATAATACTCATGTGCGG	
CECA	CAAAGTTATTTCTCCTGATCGCG	CTGCACCTTCCAATTTCTTTCC	
CECB	GCTGAAGAAGCTGGGAAAAAAG	CTTCCCAGTCCCTTGATGCC	
CECD	GAAGAAGCTGGGAAAGAAATTG	CCAATCGCTTTTATTCCTACAAC	
CECE	GAAAGCACTTCCCGTAGTAACTG	GTTAGTTATCACAATTCCCCCATG	
CECF	GTGTTCAAAGCATCGGAAAAAG	GCTGACATTCACAATCTATCTCCG	
CECG	GTTATTTCTCCTGATCGCCG	CTCGTTTTCCTGCACCTCCC	
CECH	CTTCACCAAGCTGCTATTGGT	AACTTTTTTGCCAATCTTCTTCAGC	
CECI	GGCTATTCGTTTTCGTCATTTTC	CTACGTTTTTGCCAGCCTTTTC	
CECJ	GCTATTCGTTTTCGTCATTTTTG	CTTTTCAATCTTTTTGCCCAG	
CECN	CGGCAAGAAATTGGAAAAAGTC	GAATCGATCATCCTAGGGCC	
DPT1	GCAGCATGTGGACCAATTCA	GTTCCTTCGTCCTGTTGATGG	
GAM1	GTTCCTCTTGCAAGGCATATG	GACAGTCACTGCAGCTTCTTATTG	
ATT	CAACACATTGCTGTTCACTTTCGT	TTGGAAGTTGTTACCTGGAGTGAG	
**The primers for double-strand RNA synthesis**	**Upper primer**	**Lower primer**	
AaMCR	TAATACGACTCACTATAGGGGGAGGCCGCAACACATACGAC	TAATACGACTCACTATAGGGCGATTCCCGACAAACCCACATA	
AAEL004725	TAATACGACTCACTATAGGGCCACGGGGCCTCACAAACAG	TAATACGACTCACTATAGGGCTTACAGAACGGCGGAGGATGG	
AAEL005432	TAATACGACTCACTATAGGGTTGGCCCCGACGAAATGAC	TAATACGACTCACTATAGGGACTCCGGCGCAAACACCAG	
AAEL005982	TAATACGACTCACTATAGGGTTTTGAAGGAGGCCATAAGTGC	TAATACGACTCACTATAGGGTACGCCCAAGCTCAAAACCTAC	
AAEL006355	TAATACGACTCACTATAGGGTGCGCTAGCCGGTCATTTCAT	TAATACGACTCACTATAGGGCGGCATAGTCGGGGCAACAT	
AAEL006361	TAATACGACTCACTATAGGGCGGCGCGTTTCATTTCTCC	TAATACGACTCACTATAGGGGCGGCAGTCGACTCATCAGG	
AAEL008069	TAATACGACTCACTATAGGGGCTACGGGCCGTGAAGAGTGTA	TAATACGACTCACTATAGGGTGGCTCCGATAGGCATAAGTCG	
AAEL008929	TAATACGACTCACTATAGGGGGGGCAAAGATCAACCGAACAA	TAATACGACTCACTATAGGGAAGGCCGCACGACAGGACAG	
AAEL009266	TAATACGACTCACTATAGGGGTGGGCCTCCGGGTAAGATTC	TAATACGACTCACTATAGGGTTCCCGGGTAAACGATGAGC	
AAEL012452	TAATACGACTCACTATAGGGGTGCGCCCCATCATTCAACC	TAATACGACTCACTATAGGGCTCGCATCGGCTTCCCTCAA	
AAEL014356	TAATACGACTCACTATAGGGCTGTGGCGTCGGCATAGAAGC	TAATACGACTCACTATAGGGAGGGCGCGTCCAACCATTT	
GFP	TAATACGACTCACTATAGGGGTGAGCAAGGGCGAGGAG	TAATACGACTCACTATAGGGCATGATATAGACGTTGTGGCTGTT	
DEFA	TAATACGACTCACTATAGGGGCTCGTCGATTATCACATCATTC	TAATACGACTCACTATAGGGCATTTTATAGAGCATCTAACAGTTG	
DEFC	TAATACGACTCACTATAGGGGCACGTACGATCGCCTGTC	TAATACGACTCACTATAGGGCGAGAAACTCAAAATAAACCGATAC	
DEFD	TAATACGACTCACTATAGGGCATCATTCAATTCCACAAGCTC	TAATACGACTCACTATAGGGGATCATAATAACAATGCGGC	
CECE	TAATACGACTCACTATAGGGGCAAACAGCGAACCGTAAGTCA	TAATACGACTCACTATAGGGATAGTTTTGAATATAATTTCAATGC	
CECN	TAATACGACTCACTATAGGGGCGCTCCGTCGATCAAGTTC	TAATACGACTCACTATAGGGCAGTGTCTTTCACAATTTAAATCAG	
